# UBE2M forms a positive feedback loop with estrogen receptor to drive breast cancer progression and drug resistance

**DOI:** 10.1038/s41419-024-06979-x

**Published:** 2024-08-13

**Authors:** Xiongzhi Lin, Dongsheng Sun, Shuhan Yang, Kai Cheng, XingYi Wang, Weijia Meng, Haowei Wu, Wenlin Liu, Xiaoyu Wu, Hui Yang, Xiaojun Wang, Lisha Zhou

**Affiliations:** 1 Taizhou Central Hospital (Taizhou University Hospital), School of Medicine, Taizhou University, Taizhou, Zhejiang China; 2 Graduate School of Medicine, Hebei North University, Zhangjiakou, Hebei China; 3 Department of Neurosurgery, Huashan Hospital, Fudan University, Shanghai, China

**Keywords:** Breast cancer, Neddylation

## Abstract

UBE2M, a NEDD8-conjugating enzyme, is dysregulated in various human cancers and promotes tumor cell proliferation. However, its role in estrogen receptor-positive (ER^+^) breast cancer remains unknown. We found that UBE2M expression was significantly higher in ER^+^ breast cancer tissues than in ER-negative (ER^-^) breast cancer tissues. Higher expression of UBE2M indicated a poorer prognosis in patients with ER^+^ breast cancer but not in those with ER^-^ breast cancer. Of interest, a positive feedback loop was observed between UBE2M and ERα. Specifically, ERα enhanced the HIF-1α-mediated transcription of UBE2M. In turn, UBE2M maintained ERα expression by inhibiting its ubiquitination and degradation through UBE2M-CUL3/4A-E6AP-ERα axis. Functionally, silencing of UBE2M suppressed the growth of breast cancer cells by inducing cell cycle arrest and apoptosis and improved their sensitivity to fulvestrant both in vitro and in vivo. Altogether, our findings reveal that the UBE2M-ERα feedback loop drives breast cancer progression and fulvestrant resistance, suggesting UBE2M as a viable target for endocrine therapy of ER^+^ breast cancer.

## Introduction

Breast cancer has the highest incidence and mortality rates of all cancer types among women worldwide [1]. Estrogen receptor-positive (ER^+^) breast cancer is the most common subtype and accounts for >70% of all breast cancer cases [2]. ERα, an estrogen-dependent nuclear transcription factor, plays a crucial role in the progression of breast cancer by driving the transcription of pro-survival genes and activating cellular signalling. Therefore, estrogen inhibitors and ER antagonists may serve as effective drugs for endocrine therapy in ER^+^ breast cancer [3]. Fulvestrant is a selective estrogen receptor downregulator (SERD) that targets ERα for proteasome-dependent degradation. Among several drugs used in endocrine therapy, fulvestrant is considered as the first-line drug for treating advanced or metastatic ER^+^ breast cancer in postmenopausal women [4, 5]. However, the overall survival (OS), relapse-free survival (RFS) and progression-free survival (PFS) rates remain low despite fulvestrant treatment (Clinical trials.gov, Number: NCT02422615; NCT00629616; NCT02690480), and drug resistance develops over time [6–8]. Understanding the molecular mechanisms underlying the occurrence and development of breast cancer and identifying novel therapeutic targets may help to develop strategies for improving fulvestrant sensitivity in ER^+^ breast cancer.

Neuronal precursor cell-expressed developmentally down-regulated protein 8 (NEDD8) is a ubiquitin-like protein associated with a post-translational modification, known as neddylation [9]. Similar to ubiquitination, neddylation is a process that conjugates NEDD8 to substrate proteins through three successive enzymatic cascades: NEDD8-activating enzyme E1 (NAE, a heterodimer of NAE1 and UBA3), NEDD8-conjugating enzyme E2 (UBE2M or UBE2F) and substrate-specific NEDD8-E3 ligases [10–12]. Neddylation and ubiquitination are two distinct processes, each involving its own set of E1 and E2 enzymes. The E3 enzymes involved in both processes are occasionally shared [13]. NEDD8 was initially discovered to be involved in the development of neural precursor cells [14]. Subsequent extensive studies have demonstrated that NEDD8 and enzymes of neddylation pathway are frequently overexpressed in various human cancers, including breast cancer. This overexpression is associated with disease progression and predicts poor patient survival [15–18]. The primary physiological substrates of neddylation are members of the Cullin family, such as CUL 1, 2, 3, 4 A, 4B and 5 [19]. Each Cullin protein serves as a scaffold subunit of Cullin-RING E3 ligases (CRLs), which constitute the largest family of multiunit E3 ubiquitin ligases. They control the degradation of approximately 20% of proteasome-regulated proteins and are involved in many important biological processes [20, 21]. Activation of CRLs requires the conjugation of NEDD8 to a key lysine residue at the C-terminus of Cullins. This induces a conformational change that dissociates the negative regulator CAND1 from CRLs and facilitates the assembly of functional CRLs for subsequent substrate ubiquitination [22–26]. Given that overactivation of CRLs leads to cancer growth and development, targeting the neddylation of Cullins appears to be an attractive approach for cancer treatment [27, 28]. Overactivation of neddylation pathway leads to increased neddylation modification levels of Cullins, promoting the subsequent degradation of tumor suppressors (e.g. p21 and p27) and facilitating carcinogenesis and progression [15, 16]. Additionally, targeting the overactivated neddylation enzymes suppresses tumor cell growth by inducing apoptosis, senescence or autophagy [13, 21]. Therefore, validating the neddylation pathway as a target to inactivate CRLs is a promising anticancer strategy.

Mammalian cells have two NEDD8-conjugating enzyme E2s, UBE2M and UBE2F, that play distinct and prominent roles in catalyzing neddylation. UBE2M interacts with RBX1 to regulate the neddylation of CUL 1, 2, 3, 4 A and 4B, whereas UBE2F is highly specific to the neddylation of RBX2-associated CUL 5 [29–31]. Of all neddylation enzymes, UBE2M has the strongest correlation with the level of global protein neddylation, which suggests its key role in activating the neddylation pathway [32]. Downregulation of UBE2M suppresses cancer cell proliferation by inducing cell cycle arrest, apoptosis or senescence, as demonstrated by the accumulation of multiple tumor suppressor proteins such as p21, p27 and NOXA [32–36]. UBE2M is recruited to DNA damage sites in response to ionizing radiation or other DNA-damaging agents. Therefore, depletion of UBE2M is an effective strategy for enhancing the sensitivity of cancer cells to DNA damage [37, 38]. Previous studies have demonstrated that the expression of UBE2M is elevated in multiple cancers, including breast cancer, lung cancer and esophageal squamous cell carcinoma. This upregulation is negatively associated with OS [32–35]. As mentioned above, the UBE2M-mediated neddylation pathway plays a critical role in regulating the degradation of numerous proteins. ERα is an unstable protein and undergoes ubiquitination and degradation mediated by several E3 ligases [39–42]. For that the degradation of ERα suppresses breast cancer cell growth, fulvestrant, a selective estrogen receptor downregulator, has been an effective anti-breast cancer drug. Further investigation is required to explore the involvement of UBE2M in regulating ERα expression and its role in the progression of ERα^+^ breast cancer, as well as its potential contribution to fulvestrant resistance.

In this study, high expression of UBE2M was found to be associated with a poor prognosis in patients with ER^+^ breast cancer, but not in those with ER^-^ breast cancer. Importantly, it was observed that UBE2M and ERα formed a positive feedback loop. Specifically, ERα enhanced the HIF-1α-mediated transcription of UBE2M; in turn, UBE2M maintained the expression of ERα by inhibiting its ubiquitination and degradation through the UBE2M-CUL3/4A-E6AP-ERα axis. Additionally, silencing of UBE2M suppressed the growth of breast cancer cells by inducing cell cycle arrest and apoptosis, thereby increasing the sensitivity of cancer cells to fulvestrant both in vitro and in vivo. Taken together, these findings suggest that a combination therapy involving UBE2M inhibitors and fulvestrant represents an effective therapeutic strategy for treating ER^+^ breast cancer.

## Results

### High UBE2M expression indicated a poor prognosis in ER-positive breast cancer

**Fig. 1:**
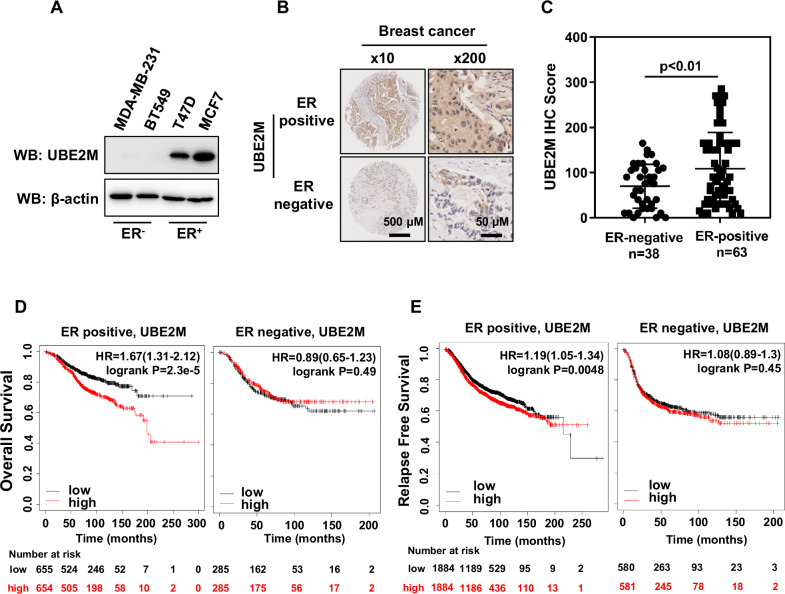
High UBE2M expression indicated a poor prognosis in ER-positive breast cancer. **A** UBE2M expression was higher in ER^+^ breast cancer cell lines than in ER^-^ breast cancer cell lines. **B**, **C** IHC analysis showed that UBE2M expression was higher in ER^+^ tumor tissues (n = 63) than in ER^-^ tumor tissues (n = 38) (scale bar for ×10 images, 500 μm; scale bar for ×200 images, 50 μm). Data are presented as mean ± SD. **p < 0.01 by two-tailed unpaired t-test. **D** Correlation between UBE2M expression and overall survival in patients with ER^+^ breast cancer or ER^-^ breast cancer (ER^+^: *p* = 2.3e-5; ER^-^: *p* = 0.49; Kaplan-Meier survival analysis). **E** Correlation between UBE2M expression and relapse-free survival in patients with ER^+^ breast cancer or ER^-^ breast cancer (ER^+^: *p* = 0.0048; ER^-^: *p* = 0.45; Kaplan-Meier survival analysis). Patients were divided into high group (high) and low group (low) according to the median of UBE2M expression. Number at risk refers to the count of individuals who are still survival at that time point.

To explore the role of UBE2M in breast cancer, we first determined its expression in two different subtypes of breast cancer cell lines (ER^+^: MCF7 and T47D cell lines; ER^-^: MDA-MB-231 and BT549 cell lines). We found that UBE2M expression was remarkably upregulated in ER^+^ breast cancer cell lines than in ER^-^ breast cancer cell lines (Fig. 1A). To further assess whether UBE2M expression relied on the ER status, the expression pattern of UBE2M was examined in 63 ER^+^ and 38 ER^-^ breast cancer tissues (n = 101) via IHC analysis. The results showed that UBE2M expression was higher in ER^+^ tumor tissues than in ER^-^ tumor tissues (*p* < 0.01) (Fig. 1B, C). In addition, we found that in 27 pairs of breast tumor tissues and normal tissues, UBE2M expression was significantly higher in breast tumor tissues than in paired adjacent normal tissues (*p* < 0.001) (Figs. S1A and S1B). Consistently, analysis of RNA-sequencing data from TCGA cohort consisting of 113 paired breast tumor tissues and adjacent normal tissues using an online software (https://www.xiantaozi.com/) validated that UBE2M expression was also upregulated in breast tumor tissues (FDR < 0.001) (Fig. S2). These results suggest that UBE2M expression is highly expressed in breast cancer and positively associated with the ER status.

Kaplan-Meier analysis (Kaplan–Meier plotter [Breast] [kmplot.com]) revealed that patients with breast cancer with higher UBE2M expression had lower OS and RFS rates than those with lower UBE2M expression (OS, *p* = 0.01, HR = 1.28; RFS, *p* = 0.0038, HR = 1.16) (Figs. S3A and S3B). It is noteworthy that the prognosis of patients with ER^+^ breast cancer with high UBE2M expression was poorer than that of patients with low UBE2M expression (OS, *p* = 2.3e-5; HR = 1.67; RFS, *p* = 0.0048, HR = 1.19) (Fig. 1D, E). However, UBE2M expression was not significantly correlated with prognosis in patients with ER^-^ breast cancer (OS, *p* = 0.49, HR = 0.89; RFS, *p* = 0.45, HR = 1.08) (Fig. 1D, E). Altogether, these findings indicate that UBE2M plays a key role in the progression of ER^+^ breast cancer.

### ERα activated the transcription of UBE2M through HIF-1α

**Fig. 2:**
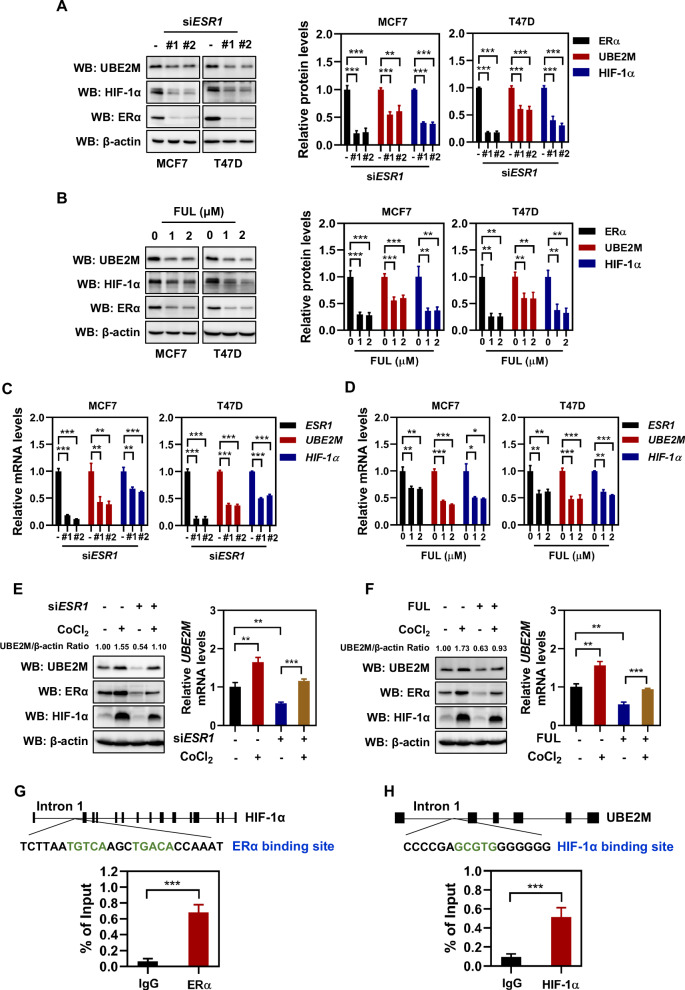
ERα transcriptionally activated UBE2M through HIF-1α. **A**, **B** Inhibition of ERα (through gene silencing or fulvestrant treatment) reduced both UBE2M and HIF-1α expression at protein levels. **C**, **D** Inhibition of ERα (through gene silencing or fulvestrant treatment) reduced both UBE2M and HIF-1α expression at mRNA levels. **E**, **F** Hypoxia restored the expression of UBE2M at both protein and mRNA levels in cells with inactivated ERα (gene silencing or fulvestrant treatment). **G** ChIP/RT-PCR was performed to assess ERα binding at the ERα binding site of HIF-1α gene. The gene structure of HIF-1α and the sequence that bears ERα binding site as indicated. The black bar represents each exon and the black line represents each intron of HIF-1α. **H** ChIP/RT-PCR was performed to assess HIF-1α binding at the HIF-1α binding site of UBE2M gene. The gene structure of UBE2M and the sequence that bears HIF-1α binding site as indicated. The black bar represents each exon and the black line represents each intron of UBE2M. Data are presented as mean ± SD. *p < 0.05, **p < 0.01, ***p < 0.001 by two-tailed unpaired t-test.

To investigate the regulatory relationship between ER and UBE2M, ERα was inhibited in MCF7 and T47D cells using siRNAs or fulvestrant treatment. Specifically, MCF7 and T47D cells were transfected with siRNA oligos targeting ESR1 for 72 hours or treated with fulvestrant for 24 hours. The results revealed that both ERα silencing and fulvestrant treatment led to a significant reduction in the expression of UBE2M at both protein and mRNA levels (Fig. 2A–D). To assess whether the downregulation of UBE2M was caused by protein degradation, we examined the effects of ERα on the half-life of UBE2M protein. As shown, the UBE2M protein was stable in MCF7 cells treated with cycloheximide (CHX), and inhibition of ERα (through gene silencing or fulvestrant treatment) did not affect its stability (Figs. S4A and S4B). These results indicate that ERα might affect UBE2M expression at the transcriptional level but not at the post-translational level.

Previous study has shown that UBE2M is transcriptionally activated by HIF-1α [43]. Indeed, we found that hypoxia (CoCl_2_ treatment or incubation in a hypoxic chamber for 24 hours) led to an increase in the mRNA and protein levels of UBE2M, accompanied by the accumulation of HIF-1α (Figs. S5A–S5D). Given that the expression of HIF-1α is high in ER^+^ breast cancer [44, 45], we speculated that ERα transcriptionally activates UBE2M through HIF-1α. To confirm this, we tested the effect of ERα on HIF-1α and found that silencing of ERα or fulvestrant treatment reduced HIF-1α expression at both protein and mRNA levels (Fig. 2A–D). However, the decrease of HIF-1α mRNA levels was relatively modest compared to the reduction in protein levels. This suggests that while ERα primarily reduces HIF-1α protein expression by inhibiting its transcription, ERα may also play a partial role in regulating HIF-1α expression at the post-transcriptional level. Here, we focused on the transcriptional regulation between ERα and HIF-1α.

To further determine the role of HIF-1α in the regulation of UBE2M by ERα, we treated ERα-silenced or fulvestrant-treated cells with 100 μM CoCl_2_ for 24 hours and found that CoCl_2_-induced hypoxia significantly restored UBE2M mRNA and protein levels (Fig. 2E, F). Analysis of the HIF-1α genome sequence, which consists of 15 exons and 14 introns, revealed that an ERα binding site is located within the first intron of HIF-1α gene (Fig. 2G). Subsequent ChIP/RT-PCR assays confirmed the ability of ERα to bind to this specific site within the HIF-1α gene (Fig. 2G). Similarly, analysis of the UBE2M genome sequence, consisting of 6 exons and 5 introns, revealed that a HIF-1α binding site is located within the first intron of UBE2M gene (Fig. 2H). MCF7 cells were treated with CoCl_2_ to activate HIF-1α and then subjected to ChIP/RT-PCR assays. The results clearly showed that binding of HIF-1α to the site was elevated compared with IgG (Fig. 2H). Therefore, these data identified HIF-1α as a direct target of ERα and UBE2M as a direct target of HIF-1α. Additionally, no ERα binding sites were found on the UBE2M genome sequence. Altogether, these findings indicate that ERα transcriptionally activates UBE2M through HIF-1α.

### UBE2M inhibited the ubiquitination and degradation of ERα via UBE2M-CUL3/4A-E6AP-ERα axis

**Fig. 3:**
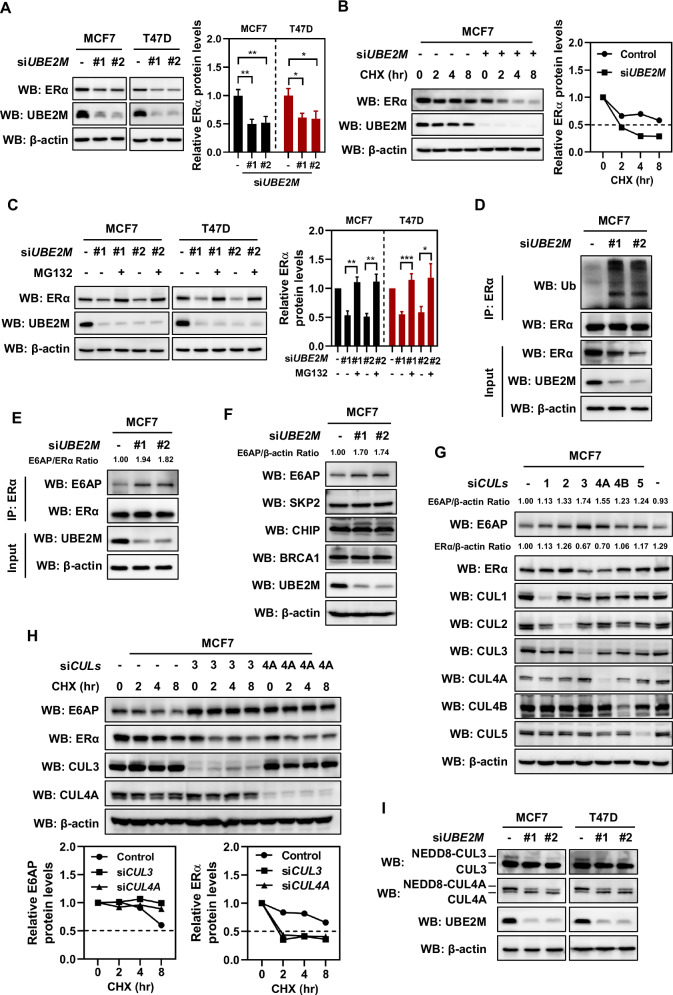
UBE2M inhibited the ubiquitination and degradation of ERα via UBE2M-CUL3/4A-E6AP-ERα axis. **A** UBE2M silencing inhibited the expression of ERα. **B** UBE2M silencing shortened the half-life of ERα protein. **C** The ERα silencing-induced decrease in UBE2M expression was reversed by the proteasome inhibitor MG132. **D** UBE2M silencing promoted the polyubiquitination of ERα. **E** UBE2M silencing promoted the interaction between ERα and E6AP. **F** UBE2M silencing enhanced the expression of E6AP. **G** CUL3 or CUL4A silencing led to elevated levels of E6AP and decreased levels of ERα. **H** CUL3 or CUL4A silencing led to an extended half-life of the E6AP protein and a shortened half-life of the ERα protein. **I** UBE2M silencing effectively inhibited neddylation levels of both CUL3 and CUL4A. Data are presented as mean ± SD. *p < 0.05, **p < 0.01, ***p < 0.001 by two-tailed unpaired t-test.

Inhibition of the NEDD8-activating enzyme E1 NAE by MLN4924 has been shown to suppress the mRNA levels of ERα [46]. Therefore, we examined whether UBE2M and ERα regulated each other’s expression through a feedback loop. Indeed, we found that silencing of UBE2M reduced the expression of ERα in ER^+^ breast cancer cell lines (Fig. 3A). Interestingly, the CHX treatment assay revealed that the half-life of UBE2M protein was significantly reduced in UBE2M-silenced MCF7 cells (Fig. 3B). Treatment with the proteasome inhibitor MG132 for 6 hours reversed the ERα silencing-induced decrease in the protein expression of UBE2M (Fig. 3C). Moreover, we found that silencing of UBE2M promoted the polyubiquitination of ERα (Fig. 3D). These results suggest that UBE2M can regulate the ubiquitination and degradation of ERα at the post-translational levels.

The ubiquitination and degradation of ERα are mediated by several E3 ligases, such as E6-associated protein (E6AP) [39, 47], carboxyl terminus of Hsp70-interacting protein (CHIP) [40, 48], breast cancer type 1 (BRCA1) [41] and S phase kinase-associated protein 2 (SKP2) [42]. Silencing of UBE2M not only promoted the interaction between ERα and E6AP but also increased the expression of E6AP (Fig. 3E, F). Based on these findings, we postulated that silencing of UBE2M may result in the accumulation of E6AP and subsequently lead to the degradation of ERα. However, given that UBE2M is a NEDD8-conjugating enzyme E2, it should not directly regulate ubiquitination and protein stability of E6AP and ERα. Instead, it most likely functions by enhancing the neddylation levels of one or more CULs to activate CRLs, which, as the largest family of multiunit E3 ubiquitin ligases, control degradation of about 20% of proteasome-regulated proteins [20, 21]. To this end, we individually silenced Cullins in MCF7 cells and observed that among all six Cullins, silencing of CUL3 or CUL4A resulted in elevated levels of E6AP, while silencing of these two Cullins led to decreased levels of ERα (Fig. 3G). Furthermore, silencing of CUL3 or CUL4B led to an extended half-life of the E6AP protein and a shortened half-life of the ERα protein (Fig. 3H). Additionally, western blotting analysis confirmed that silencing UBE2M effectively inhibited neddylation levels of both CUL3 and CUL4A (Figs. 3I and S6). Thus, through inhibiting the neddylation levels of CUL3 and CUL4A to further inactivate these two CRLs, silencing of UBE2M can effectively inhibit the protein degradation of E6AP, thereby inducing ERα ubiquitination and degradation. These findings collectively suggest that UBE2M plays a crucial role in promoting ERα expression by blocking its ubiquitination and degradation, which is mediated by UBE2M-CUL3/4A-E6AP-ERα axis.

### UBE2M silencing inhibited cell growth by inducing cell cycle arrest and apoptosis

**Fig. 4:**
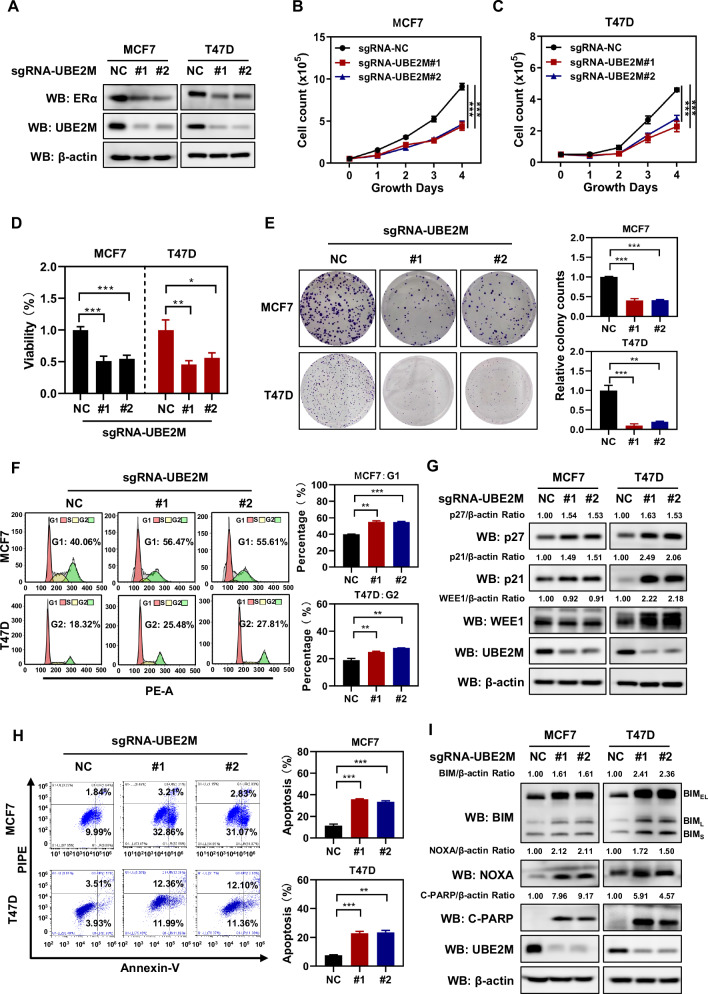
UBE2M silencing inhibited cell growth by inducing cell cycle arrest and apoptosis. **A** Two sgRNA-UBE2M oligos were used to silence UBE2M in MCF7 and T47D cells using the CRISPR/Cas9 system. **B**–**D** UBE2M silencing inhibited the growth of both MCF7 and T47D cells. **E** UBE2M silencing inhibited the colony-forming ability of both MCF7 and T47D cells. **F**, **G** UBE2M silencing induced cell cycle arrest. PI staining and FACS analysis were used to analyze the cell cycle profile upon UBE2M silencing (**F**). Western blotting was used to assess the expression of cell cycle-related proteins, namely, p21, p27 and WEE1 (**G**). **H, I** UBE2M silencing induced cell apoptosis. AnnexinV-FITC/PI double-staining analysis was used to analyze the cell apoptosis upon UBE2M silencing (H). Western blotting was used to assess the expression of the apoptosis-related proteins BIM (BIM_EL_, BIM_L_ and BIM_S_), NOXA and cleaved PARP (C-PARP) (**I**). Data are presented as mean ± SD. *p < 0.05, **p < 0.01, ***p < 0.001 by two-tailed unpaired t-test.

Given that UBE2M is considered as an oncogene [32, 33], we validated its potential as a therapeutic target for ERα^+^ breast cancer. Stable MCF7 and T47D cells with UBE2M silencing were generated using the CRISPR/Cas9 system. The silencing efficiency was validated via western blotting. The results showed that silencing of UBE2M decreased the expression of ERα (Fig. 4A). Cell proliferation and ATP-lite luminescence assays showed that silencing of UBE2M suppressed the proliferation of both MCF7 and T47D cells (Fig. 4B–D). In addition, clonogenic assay showed that silencing of UBE2M significantly suppressed the colony-forming ability of both cell lines (Fig. 4E).

Cancer cell growth is suppressed in part through cell cycle arrest and apoptosis. Silencing of UBE2M remarkably led to cell cycle arrest at different stages in a cell line-dependent manner. As evidenced by the upregulation of the G1 phase arrest markers p27 and p21, silencing of UBE2M increased the proportion of MCF7 cells in the G1 phase (Fig. 4F, G). As evidenced by the upregulation of the G2 phase arrest marker WEE1, silencing of UBE2M increased the proportion of T47D cells in the G2 phase (Fig. 4F, G). In addition, silencing of UBE2M triggered apoptosis in both cell lines as evidenced by the significant increase in the number of Annexin V-positive cells, and the accumulation of apoptosis-related proteins NOXA and cleavage of PARP (Fig. 4H, I). Moreover, we found that silencing of UBE2M resulted in an upregulation of BIM, an apoptotic protein that has recently been reported to mediate UBE2M deletion-induced apoptosis in double-negative T cells [49] (Fig. 4I). Altogether, these results indicate that silencing of UBE2M suppresses the growth of ER^+^ breast cancer cells by inducing cell cycle arrest and apoptosis. As a result, UBE2M may serve as a promising therapeutic target for ER^+^ breast cancer.

### UBE2M silencing sensitized ER^+^ breast cancer cells to fulvestrant in vitro and in vivo

**Fig. 5:**
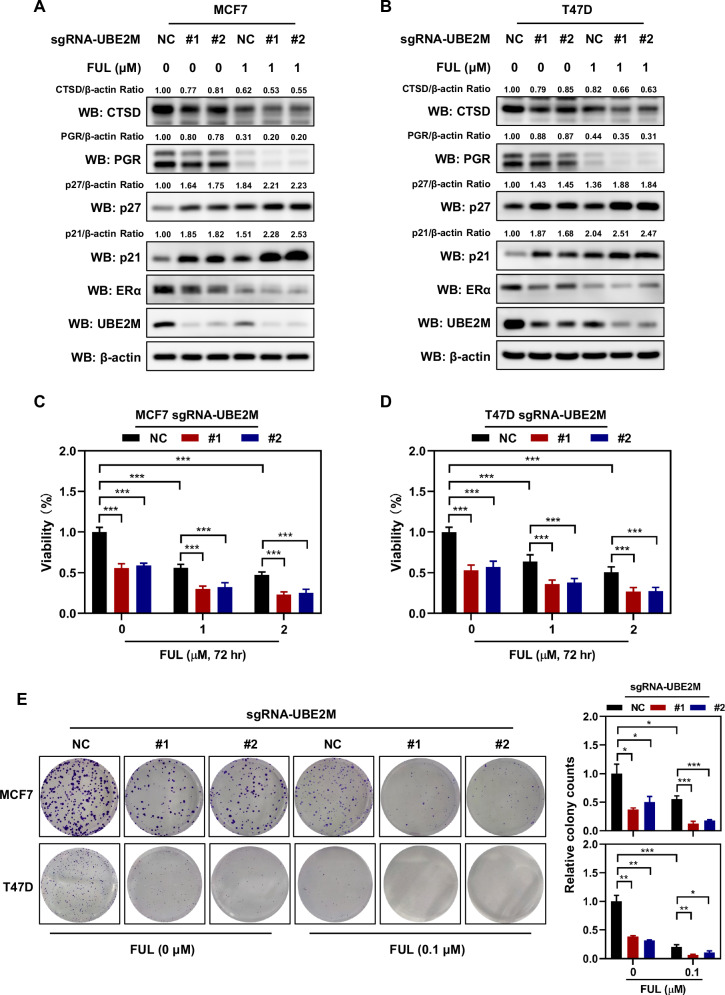
UBE2M silencing sensitised ER-positive breast cancer cells to fulvestrant in vitro. **A**, **B** UBE2M silencing and fulvestrant synergically inhibited the expression of ERα and UBE2M. **C**, **D** UBE2M silencing and fulvestrant synergically inhibited cell growth. The cell growth was determined via ATP-lite assay. **E** UBE2M silencing and fulvestrant synergically inhibited the colony-forming ability of breast cancer cells. The colony-forming ability was determined via colony formation assay. Data are presented as mean ± SD. *p < 0.05, **p < 0.01, ***p < 0.001 by two-tailed unpaired t-test.

Considering the positive feedback regulation between ERα and UBE2M, we hypothesised that UBE2M might be associated with the development of fulvestrant resistance. To verify this hypothesis, MCF7 and T47D cells were treated with fulvestrant and subjected to UBE2M silencing. The results showed that UBE2M silencing and fulvestrant treatment synergically inhibited the expression of ERα and UBE2M (Fig. 5A and B). Consistently, the combination of UBE2M silencing and fulvestrant synergistically inhibited two downstream substrates of ERα, namely, progesterone receptor (PGR) and cathepsin D (CTSD), and induced the expression of p27 and p21, which were accumulated upon inactivation of neddylation (Fig. 5A, B). In addition, the combination of UBE2M silencing and fulvestrant suppressed the proliferation and colony-forming ability of MCF7 and T47D cells (Fig. 5C–E).

**Fig. 6:**
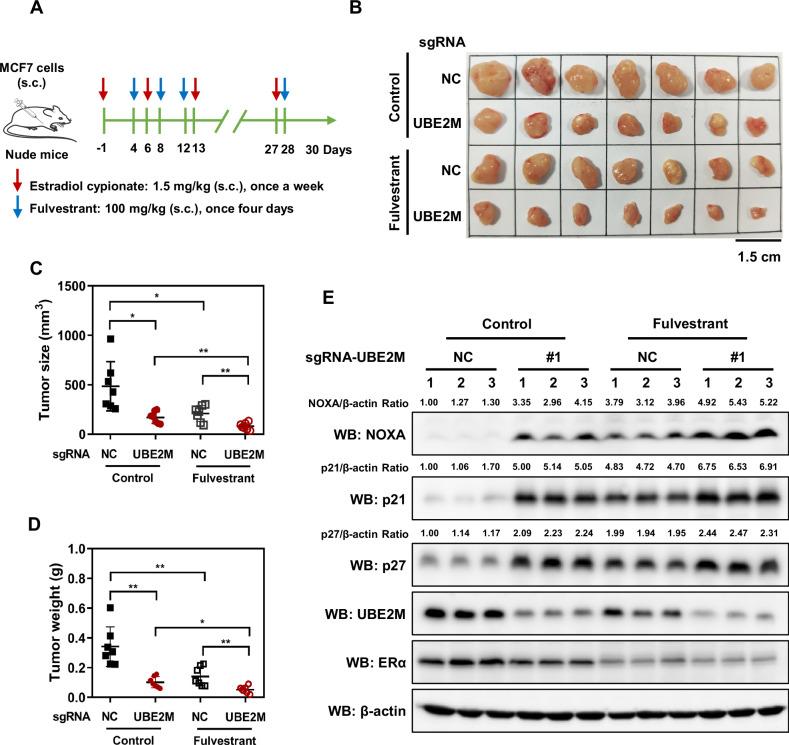
UBE2M silencing sensitised ER-positive breast cancer cells to fulvestrant in vivo. **A** Generation of subcutaneous tumor models. Estradiol cypionate (1.5 mg/kg, once a week) was subcutaneously (s.c.) injected into each mouse (5-week-old female nude mice) 1 day before the injection of MCF7 cells (day −1). A total of 1 × 10^7^ stable cells with matrigel (1:1) were subcutaneously injected into the right armpits of nude mice (day 1). Thereafter, the mice were subcutaneously injected with corn oil or fulvestrant (day 4) (100 mg/kg, once every 4 days). Each group comprises 7 mice (n = 7). **B** Tumors as described in (**A**) were excised and photographed (scale bar = 1.5 cm). **C** UBE2M silencing and fulvestrant synergically decreased tumor volume. **D** UBE2M silencing and fulvestrant synergically decreased tumor weight. **E** UBE2M silencing and fulvestrant synergically promoted cell cycle arrest and apoptosis. Data are presented as mean ± SD. *p < 0.05, **p < 0.01 by two-tailed unpaired t-test.

To verify the effects of UBE2M silencing on fulvestrant sensitivity in vivo, we established subcutaneous tumor models using MCF7 cells (Fig. 6A). Silencing of UBE2M significantly inhibited tumor growth, volume and weight in nude mice with subcutaneous implantation of MCF7 cells (Fig. 6B–D). Compared with UBE2M silencing or fulvestrant treatment alone, the combination of UBE2M silencing and fulvestrant inhibited tumor growth more significantly, suggesting that UBE2M silencing improved the sensitivity of ER^+^ breast cancer cells to fulvestrant (Fig. 6B–D). Consistently, the combination of UBE2M silencing and fulvestrant increased the accumulation of the cell cycle inhibitors p21 and p27 and the pro-apoptotic protein NOXA in tumor cells (Fig. 6E). Altogether, the results indicate that UBE2M silencing increases the sensitivity of ER^+^ breast cancer cells to fulvestrant by inducing cell cycle arrest and apoptosis, suggesting that UBE2M is a potential driver of fulvestrant resistance.

## Discussion

In this study, we followed up our observation that UBE2M and ERα formed a positive feedback loop. Mechanistically, ERα enhanced the HIF-1α-mediated transcription of UBE2M (Fig. 2). In turn, UBE2M maintained ERα expression by inhibiting its ubiquitination and degradation through UBE2M-CUL3/4A-E6AP-ERα axis (Fig. 3). Additionally, silencing of UBE2M suppressed the growth of breast cancer cells by inducing cell cycle arrest and apoptosis (Fig. 4), thereby increasing the sensitivity of cancer cells to fulvestrant both in vitro and in vivo (Figs. 5 and 6). Collectively, these findings indicate that UBE2M plays a crucial role as an oncogene and that the combination of its inhibitor with fulvestrant presents as an effective treatment strategy for ER^+^ breast cancer.

One important finding of this study is that ERα and UBE2M function through a positive feedback loop. In this loop, ERα enhanced the transcription of UBE2M (Fig. 2), while UBE2M, in turn, maintained ERα expression by regulating its ubiquitination and degradation (Fig. 3). How does UBE2M, a NEDD8-conjugating enzyme E2, not an E3, regulate ERα ubiquitination and degradation? Although a recent report has indicated that UBE2M may undergo auto-neddylation [50], its primary known biochemical function is to activate CULs 1-4 as an E2 [29, 30]. Our observation that silencing of UBE2M decreased ERα and correspondingly increased its classic E3 ubiquitin ligase E6AP, strongly suggests us to determine the relationship between CULs and E6AP. In CULs 1-5, both CUL3 and CUL4A can negatively regulate E6AP protein levels by shortening its half-life; however, they can also positively regulate ERα protein levels by extending its half-life (Fig. 3). E6AP is a classic E3 ubiquitin ligase for ERα [39, 47], which further support the notion that UBE2M promoted neddylation levels of CUL3/4 A to activate CRL3/4 A and subsequently led to the degradation of E6AP. This reduction in E6AP then inhibited the ubiquitination and degradation of ERα and resulted in its accumulation. Thus, E6AP may be a novel substrate of CRL3 or CRL4A ubiquitin ligase. Although neddylation and ubiquitination modifications share similarities in their processes, there is currently no evidence supporting direct regulation of protein degradation through neddylation. Thus, we established regulatory axis between neddylation and ubiquitination, namely UBE2M-CUL3/4A-E6AP-ERα axis. Additionally, our finding showed that UBE2M also disrupted the interaction between ERα and E6AP, thereby inhibiting the ubiquitination and degradation of ERα. A previous study reported that phosphorylation of ERα at Ser^294^ promotes its binding to E6AP, resulting in the degradation of ERα [39]. In addition to upregulating E6AP expression to facilitate the binding of ERα and E6AP, silencing of UBE2M may also enhance the interaction between ERα and E6AP by modulating the phosphorylation of ERα Ser^294^, thereby promoting the degradation of ERα.

ERα is a classical transcription factor [51], however, no ERα binding sites were identified on the UBE2M genome sequence. This raises the question of how ERα transcriptionally activate UBE2M. Previous studies have demonstrated the presence of a HIF-1α binding site on the UBE2M genome sequence, and UBE2M is transcriptionally activated by HIF-1α in response to hypoxia [43]. Moreover, HIF-1α contains ERα-binding elements, and ERα knockdown directly reduces HIF-1α expression [52]. Our findings are consistent with these conclusions. Upon these observations, we formulated a working hypothesis to investigate whether ERα enhances the transcription of UBE2M mediated by HIF-1α. Indeed, CoCl_2_-induced HIF-1α was found to reverse the ERα silencing-induced decrease in the mRNA expression of UBE2M (Fig. 2). Thus, we made a novel observation that ERα transcriptionally activates UBE2M through HIF-1α, suggesting that overexpression of UBE2M may be attributed to the high expression of HIF-1α in ER^+^ breast cancer [44, 45]. Collectively, these findings in our study demonstrate that the ERα/HIF-1α/UBE2M axis is an oncogenic cascade regulating the development of breast cancer. However, we also found that HIF-1α expression was higher in ER^-^ breast cancer cell lines compared to ER^+^ breast cancer cell lines (Fig. S7). The expression patterns of HIF-1α and UBE2M were opposite in these two types of breast cancer cell lines. This observation may be attributed to the fact that ER^-^ tumors exhibit a greater proliferation rate and are more hypoxic than ER^+^ tumors, leading to increased HIF-1α expression. Kaplan-Meier analysis revealed that ER^+^ breast cancer patients with high HIF-1α expression had a worse prognosis compared to those with low HIF-1α expression; while, in ER^-^ breast cancer patients, HIF-1α expression was weakly associated with prognosis (Fig. S8). These findings indicate that the regulatory mechanism of HIF-1α may differ between ER^+^ and ER^-^ breast cancer. There may be additional potential mechanisms, independent of HIF-1α, that regulate the expression of UBE2M in ER^-^ breast cancer cells. The focus of this study is to investigate the regulatory mechanism of UBE2M expression in ER^+^ breast cancer, which is mediated by HIF-1α.

Increasing lines of evidence have strongly suggested that UBE2M acts as an oncogene, which is altered in over two-thirds of all human cancers [30]. Here, we reported that silencing of UBE2M significantly suppressed the growth of ER^+^ breast cancer cells by inducing cell cycle arrest and apoptosis (Fig. 4), which is consistent with the aforementioned results. Upon thorough investigation of the effects of UBE2M on fulvestrant resistance in breast cancer, we found the following results: (a) The combination of UBE2M silencing and fulvestrant treatment synergistically inhibited the downstream signalling pathways of both ERα and UBE2M (Fig. 5); (b) The combination of UBE2M silencing and fulvestrant treatment synergistically inhibited the growth of breast cancer cells both in vitro and in vivo (Figs. 5 and 6). MLN4924, a NAE inhibitor, has been reported to synergize with fulvestrant in inhibiting the growth of breast cancer cells [46]. However, tumor cells are prone to forming UBA3 mutations, leading to MLN4924 resistance [53, 54]. These findings further support that the ERα-UBE2M positive feedback loop is a promising therapeutic target for breast cancer. It is also crucial to note that in order to assess the effect of UBE2M on the fulvestrant resistance of breast cancer cells, it would be preferable to utilize cell lines derived from fulvestrant-resistant patients.

**Fig. 7:**
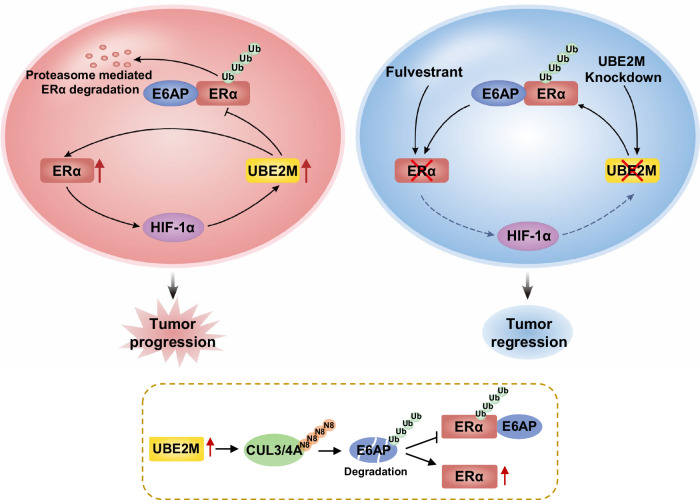
A proposed working model for the tumor-promoting role of the ERα-UBE2M positive feedback loop in ERα-positive breast cancer. UBE2M forms a positive feedback loop with ERα. ERα enhances the HIF-1α-mediated transcription of UBE2M, and UBE2M in turn maintains ERα expression via UBE2M-CUL3/4A-E6AP-ERα axis. Silencing of UBE2M suppresses the growth of breast cancer cells by inducing cell cycle arrest and apoptosis and improves their sensitivity to fulvestrant both in vitro and in vivo.

In summary, our study supports the following model: UBE2M, a NEDD8-conjugating enzyme E2, forms a positive feedback loop with ERα. Specifically, ERα enhances the HIF-1α-mediated transcription of UBE2M; in turn, UBE2M maintains ERα expression by inhibiting its ubiquitination and degradation through UBE2M-CUL3/4A-E6AP-ERα axis. Silencing of UBE2M suppresses the growth of breast cancer cells by inducing cell cycle arrest and apoptosis and enhances their sensitivity to fulvestrant both in vitro and in vivo (Fig. 7). These findings indicate that the UBE2M-ERα positive feedback loop drives tumor progression and drug resistance in breast cancer. Therefore, targeting UBE2M represents a promising strategy for improving the sensitivity of ER^+^ breast cancer to endocrine therapy.

## Materials and Methods

### Generation of stable UBE2M-knockdown cell lines using CRISPR/Cas9

UBE2M-knockdown cells were generated using CRISPR-Cas9 technology as previously described [55, 56]. Briefly, small guide RNAs (sgRNAs) were individually designed to target the human UBE2M gene, utilizing the online design tool available at crispr.mit.edu. Complementary sgRNA oligo DNAs were synthesized by Sangon Biotech (Shanghai, China), annealed to form double-strand DNA and cloned into a BsmB I restriction enzyme digested lenti-Guide-CRISPR-v2-puro vector. The cloned fragments were DNA sequenced to confirm their fidelity. The lenti-Guide-CRISPR-v2-puro vector containing nontarget control (NC) gRNA or lenti-Guide-CRISPR-v2-puro vector containing UBE2M gRNA (4.0 μg) was co-transfected with psPAX2 (3.0 μg) and pMD2.G plasmids (1.0 μg) into HEK293T cell (80% density in 10 cm dish) using Lipofectamine 3000 (Invitrogen, USA) according to the manufacturer’s recommendations. After 48 hours of transfection, the viral supernatants were harvested, filtered and mixed with 10 μg/mL polybrene (Beyotime, C0351) to enhance the infection efficiency. MCF7 and T47D cells at 40% density in six-well plates were infected with about 10^6^ titer of virus for 48 hours. Infected cells were selected with 10 μg/mL puromycin (Beyotime, ST551) for 5 days, and then collected to determine knockdown effect by western blotting. The sequence of NC oligo: 5´-AAGAAGAATTGGGGATGATG-3´. The sequences of human sgRNA oligos targeting UBE2M#1: 5´-TCACCAAGAAGAGATACTGC-3´ and UBE2M#2: 5´-AGATGACCAGCTTGAAGTTG-3´.

### Chromatin immunoprecipitation (ChIP)/RT-PCR assay

ChIP assay was performed with 10^6^ cells using ChIP Assay Kit (Beyotime, P2078) according to the manufacturer’s instructions. Briefly, cells in a 10 cm dish were fixed with 1% formaldehyde (10 mL medium + 270 μL fresh 37% formaldehyde) at 37 °C for 10 minutes and then quenched with 0.125 M glycine at room temperature for 5 minutes. The medium was removed, and the cells were washed twice with 10 mL of ice-cold PBS containing 1 mM PMSF, ensuring complete removal of the wash from the culture dish each time. The cells were then scraped off in 1 mL of ice-cold PBS containing 1 mM PMSF, counted, and centrifuged at 1,000 g for 2 minutes (10^6^ cells). The cell pellet was suspended in 0.2 mL lysis buffer supplemented with 1 mM PMSF for a further incubation of 10 minutes at 4 °C. Chromatin was sonicated to obtain DNA fragments with an average length of 200 to 1,000 bp (50 W, 10 s work/10 s stop, 10 cycles). A soluble fraction of sheared chromatin was diluted with 1.8 mL ChIP dilution buffer containing 1 mM PMSF. 20 μL sample was taken as input, and the rest was incubated with IgG, ERα or HIF-1α antibody overnight at 4 °C with rotation. 60 μL Protein A + G Agarose/Salmon Sperm DNA were then added to each immunoprecipitation reaction and incubated for 1 hours at 4 °C with rotation. Chromatin-captured beads were collected by at 1000 g for 1 minute, and washed by low-salt wash (once), high-salt wash (once), LiCl wash (once), and TE buffer (twice) at 4 °C for 5 minutes with rotation. Beads were resuspended in 250 uL elution buffer (1% SDS, 0.1 M NaHCO_3_) for 5 minutes at room temperature and then centrifuged at 1,000 g for 1 minute. The 250 uL supernatant was carefully collected. Subsequently, DNA was reverse cross-linked by adding 10 μL 5 M NaCl at 65 °C for 4 hours, followed by an addition of 5 μL 0.5 M EDTA, 10 μL 1 M Tris (pH 6.5) and 0.5 μL 20 mg/mL protease K at 45 °C for 1 hour. DNA purification kit (Beyotime, D0033) was used to purify DNA. Finally, the purified DNA was used to perform RT-PCR. 1% of the initial chromatin was utilized as input. The percent input method was employed to standardize the data. The primers used for PCR were synthesized by Sangon Biotech (Sangon Biotech, China), and their sequences were as follows: HIF-1α-Intron 1-ERα binding forward: 5´-ATTGAATGTTTGCTGGAACG-3´; HIF-1α-Intron 1-ERα binding reverse: 5´-TTCCATAAGAGAATTCAGTTACTGTTC-3´; UBE2M-Intron 1-HIF-1α binding forward: 5´-TGGGAGTCAGGAAGGGGTAAT-3´; UBE2M-Intron 1-HIF-1α binding reverse: 5´-AGTCCCTAGTAGCAGACACC-3´.

### Cell proliferation and colony formation assays

For cell proliferation assay, cells were seeded in 6-well plates at a density of 5 × 10^4^ cells/well. The culture medium was replaced and cells were counted every day for 4 consecutive days using cell counter (BIO-RAD TC20, USA). For ATPlite luminescence assay, cells were seeded in 96-well plates at a density of 2000 cells/well and cultured for 72 hours. Cell proliferation was determined using an ATPlite luminescence assay kit (PerkinElmer, USA) according to the manufacturer’s protocol. For colony formation assay, cells were seeded in 6-well plates at a density of 500 cells (MCF7) or 1,000 cells (T47D) per well and cultured at 37°C for 10 (MCF7) or 14 (T47D) days. The resulting cell colonies were fixed with 4% paraformaldehyde for 20 minutes and stained with 0.1% crystal violet solution for 30 minutes. Subsequently, colonies with more than 50 cells were counted. The experiments were performed in triplicate.

### Flow cytometric analysis of the cell cycle and apoptosis

Cells were digested with 0.25% trypsin without EDTA, and the digestion was terminated by DMEM medium containing 10% FBS. Subsequently, cells were collected and centrifuged at 1,200 rpm for 5 minutes, followed by two washes with cold PBS. For cell cycle analysis, a total of 5×10^5^ cells were fixed with 1 mL ice-cold 70% ethanol at -20°C overnight. After centrifugation at 1,200 rpm for 5 minutes and two washes with cold PBS buffer, cells were suspended in 200 μL cold PBS buffer containing 50 ug/ml propidium iodide (PI, Beyotime, ST1569) and 10 μg/mL RNase A (Beyotime, ST578). Following a dark incubation of 15 minutes at room temperature, cells were used for cell cycle analysis using Flow cytometry (Beckman Coulter, USA). For apoptosis assays, the Annexin V-FITC Apoptosis Detection Kit (Beyotime, C1062L) was used according to the manufacturer’s recommendations. A total of 1×10^5^ cells were suspended in 195 μL Annexin V-FITC binding solution containing 5 μL Annexin V-fluorescein isothiocyanate (FITC) and 10 μL PI. Following a dark incubation of 15 minutes at room temperature, cells were subjected to flow cytometric analysis. The voltage and the fluorescence compensation were needed to be adjusted. Data from flow cytometry were analysed using Kaluza Analysis software. The experiments were performed in triplicate.

### Immunohistochemical analysis of human breast cancer tissue arrays

Tissue microarray of human breast cancer and adjacent normal tissues were purchased from Shanghai Outdo Biotech Company (Shanghai, China). This study was approved by the Ethics Committee of Shanghai Outdo Biotech Company (approval no.: SHYJS-CP-1304003; Shanghai, China). All patients provided written informed consent prior to inclusion in the study.

For immunohistochemical analysis, human breast cancer tissue array sections were de-paraffinized and then treated with a 3% hydrogen peroxide solution to block endogenous peroxidases. Antigen recovery was achieved by using 0.1 M sodium citrate buffer (pH 6.0) for one hour. Subsequently, the array sections were incubated with anti-UBE2M antibody (Abcam, 109507, diluted at 1:1000) at 4 °C overnight, followed by the sequential addition of a secondary antibody conjugated to horseradish peroxidase and a diaminobenzidine substrate. The array sections were then counterstained with hematoxylin, dehydrated, and examined using a photomicroscope Aperio ScanScope XT (Leica Microsystems, Inc.).

For histological evaluation, the tissue sections were semi-quantitatively evaluated based on the percentage of positively stained tumor cells and the grade of staining intensity as described previously [57, 58]. The staining intensity of breast cancer cells was divided into 4 grades as follows: no staining = 0, weak staining = 1, moderate staining = 2 and strong staining = 3. The percentage of positive cells was calculated and scored from 0 to 100%. Then, the proportion and intensity were multiplied to produce a total score of 0 through 300 (0: 0% of cells stained; 300: 100% of cells with strong staining intensity). IHC scores (proportion and intensity) were shown in Supplementary Tables 1 and 2.

### Subcutaneous tumor model

As previously mentioned, UBE2M knockdown MCF7 cell lines were generated using the CRISPR/Cas9 system. Long-term screening was performed with a low concentration of 1 μg/mL of purinomycin. The knockdown effect of UEB2M in MCF7 cell line before subcutaneous injection was detected by western blotting. Female nude mice (nu/nu, 5 weeks old, weight approximately 16 g, n = 7) were purchased from Charles River (Shanghai, China). All mice were randomly grouped before further experiments. Estradiol cypionate (Selleck, 313-06-4) (1.5 mg/kg, once a week) was subcutaneously injected into each mouse 1 day before the injection of MCF7 cells. A total of 1 × 10^7^ MCF7 cells (with sgRNA-NC or -UBE2M) supplemented with matrigel (Corning, 354248) (1:1) were subcutaneously injected into the right armpits of nude mice, followed by subcutaneous injection of corn oil or fulvestrant (Selleck, S1191; 100 mg/kg, once every 4 days). Tumor size was measured using a vernier calliper and calculated using the formula volume: (length × width^2^) / 2. Tumor weight was measured using an analytical balance. Animal studies were performed in accordance with animal protocol procedures and approved by the Medical Ethics Committee of Taizhou College of Medicine (approval no.: TZYXY2020-005; Taizhou, China). No blinding was performed for the animal experiments.

### Statistical analysis

Data were presented as the mean ± standard deviation (SD) from at least three independent experiments. Statistical analysis was performed using the GraphPad Prism 5 software (Graph Pad Software, USA). The two-tailed unpaired t-test was used to assess the differences between the two groups [59, 60]. In brief, the F-test was first used to compare the variance between the two groups. If the *p*-value of the F-test ≥ 0.05, equal variance is assumed when performing the unpaired t-test. If the *p*-value of the F-test < 0.05, variance is not assumed equally and thus Welch’s correction is applied for unpaired t-test. Wilcoxon signed-rank test in R was used for the analysis of RNA-sequencing data from a TCGA dataset. Adjusted *p*-values were calculated based on the *p*-values to control the False Discovery Rate (FDR) using the Benjamini-Hochberg (BH) method, implemented via the *p*. adjust function with the method set to “BH”. Three levels of significance were defined for all tests: *p < 0.05, **p < 0.01, ***p < 0.001. The sample sizes were determined based on previous experience in the laboratory and pre-specified effect sizes considered to be biologically significant. No samples or animals were excluded from any analyses. Blind analysis was not performed in this study.

### Supplementary information

Supplementary Figures and Methods

Supplementary Table 1

Supplementary Table 2

Supplementary Table 3

Supplementary Table 4

Original Western Blots

## Data Availability

The data that support the findings of this study are included in this article. Additional data related to this study are available on request from the corresponding author.
