# Supplementary material for: UBE2M forms a positive feedback loop with estrogen receptor to drive breast cancer progression and drug resistance
Source: Cell Death Dis. 2024 Aug 13;15(8):590. doi: 10.1038/s41419-024-06979-x (PMC11322533; doi:10.1038/s41419-024-06979-x)
Supplement: Supplementary file 1 — Supplementary Figures and Methods [file 41419_2024_6979_MOESM1_ESM.pdf]

# Supplementary Figures and Methods

## Supplementary Figure 1

**Analysis of IHC data showed that the protein expression of UBE2M was higher in breast cancer tissues than in normal tissues**

## Supplementary Figure 2

**Analysis of RNA-sequencing data from a TCGA dataset showed that the mRNA expression of UBE2M was higher in breast cancer tissues than in normal tissues**

## Supplementary Figure 3

**Correlation between UBE2M expression and overall survival and relapse-free survival in patients with all types of breast cancer**

## Supplementary Figure 4

**Inhibition of ERα (through gene silencing or fulvestrant treatment) did not affect the stability of UBE2M protein**

## Supplementary Figure 5

**Hypoxia ($CoCl_2$ treatment or incubation in a hypoxic chamber) led to the accumulation of UBE2M at both protein and mRNA levels**

## Supplementary Figure 6

**The effect of UBE2M silencing on neddylation levels of CULs.**

## Supplementary Figure 7

**The expression of HIF-1α in $ER^+$ and $ER^-$ breast cancer cell lines.**

## Supplementary Figure 8

**Correlation between HIF-1α expression and overall survival and relapse-free survival in breast cancer patients with different ER status.**

## Supplementary Materials and Methods

# Lin et al. Supplemental Figure 1

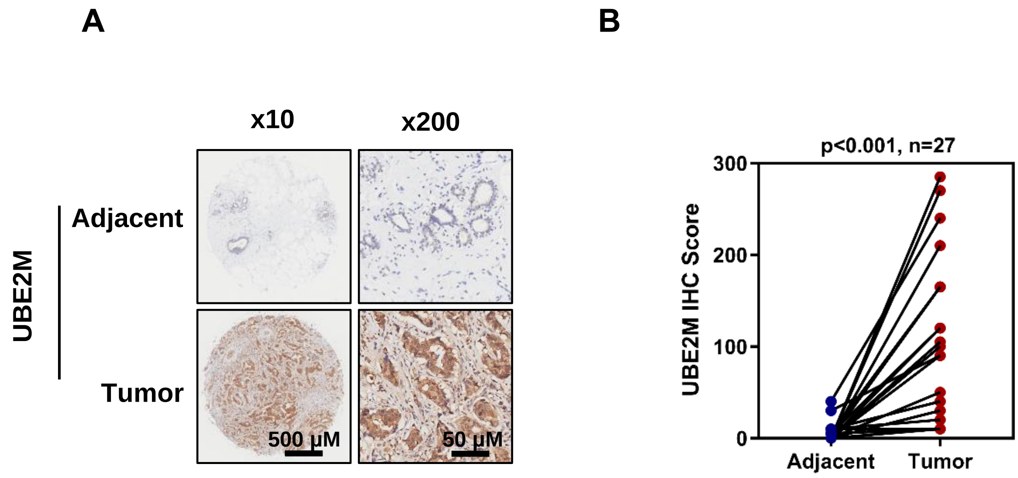


**Figure S1. Analysis of IHC data showed that the protein expression of UBE2M was higher in breast cancer tissues than in normal tissues.** (scale bar for ×10 images, 500 µm; scale bar for ×200 images, 50 µm) (****p* $< 0.001$; paired t-test, n = 27).

# Lin et al. Supplemental Figure 2

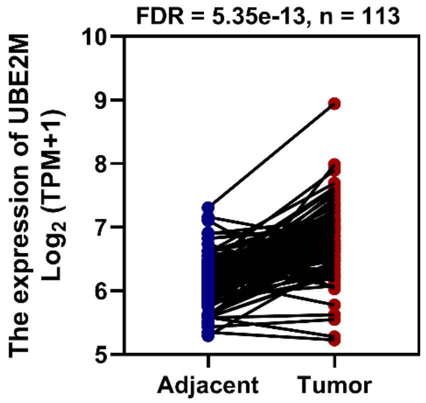


**Figure S2. Analysis of RNA-sequencing data from a TCGA dataset showed that the mRNA expression of UBE2M was higher in breast cancer tissues than in normal tissues.** The RNA-sequencing data were shown in Supplementary Table 3.

# Lin et al. Supplemental Figure 3

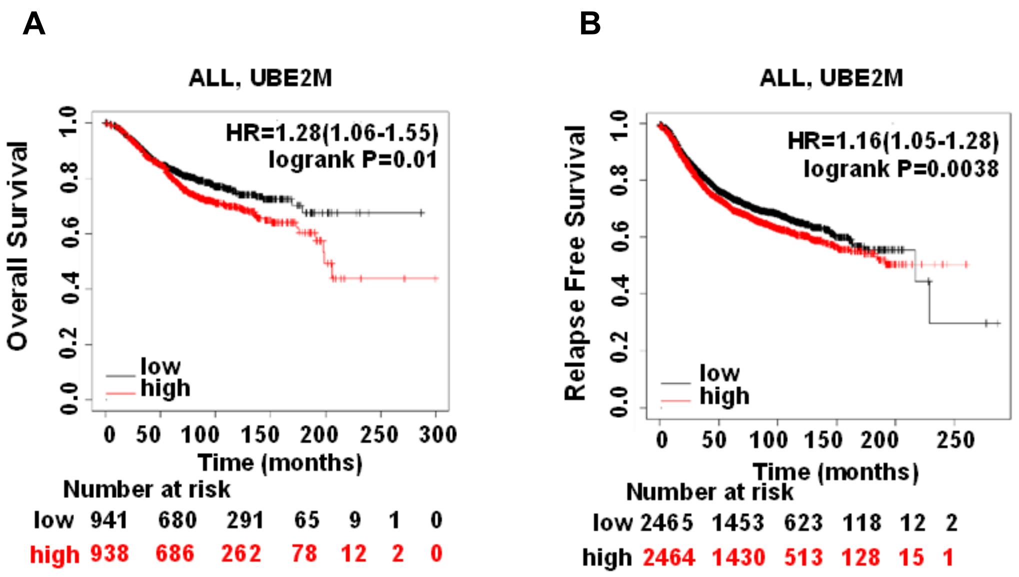


**Figure S3. Correlation between UBE2M expression and overall survival and relapse-free survival in patients with all types of breast cancer**. Patients were divided into high group (high) and low group (low) according to the median of UBE2M expression. Number at risk refers to the count of individuals who are still survival at that time point.

# Lin et al. Supplemental Figure 4

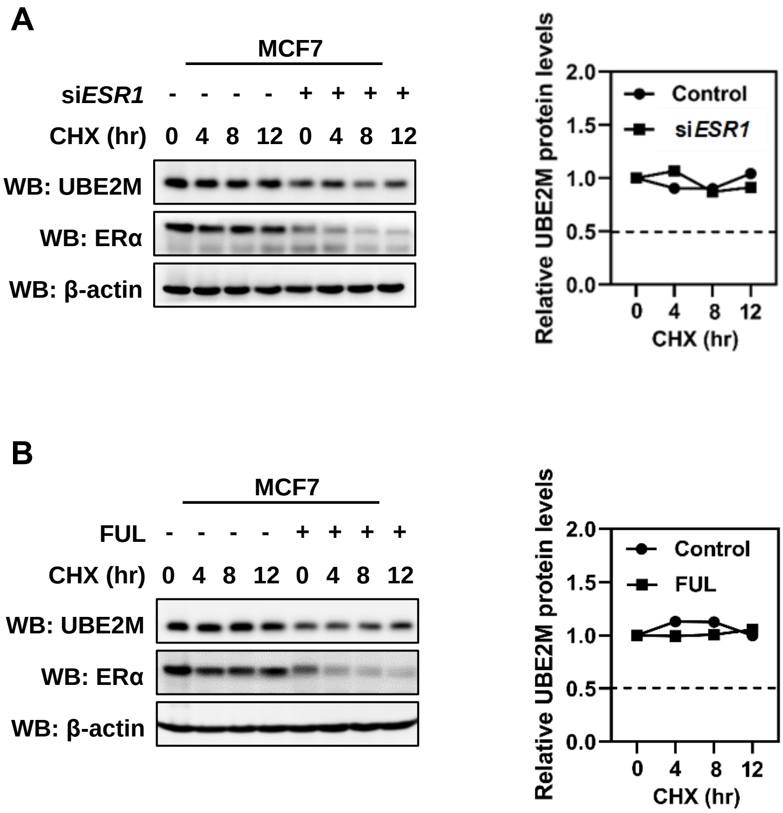


**Figure S4. Inhibition of ERα (through gene silencing or fulvestrant treatment) did not affect the stability of UBE2M protein.**

# Lin et al. Supplemental Figure 5

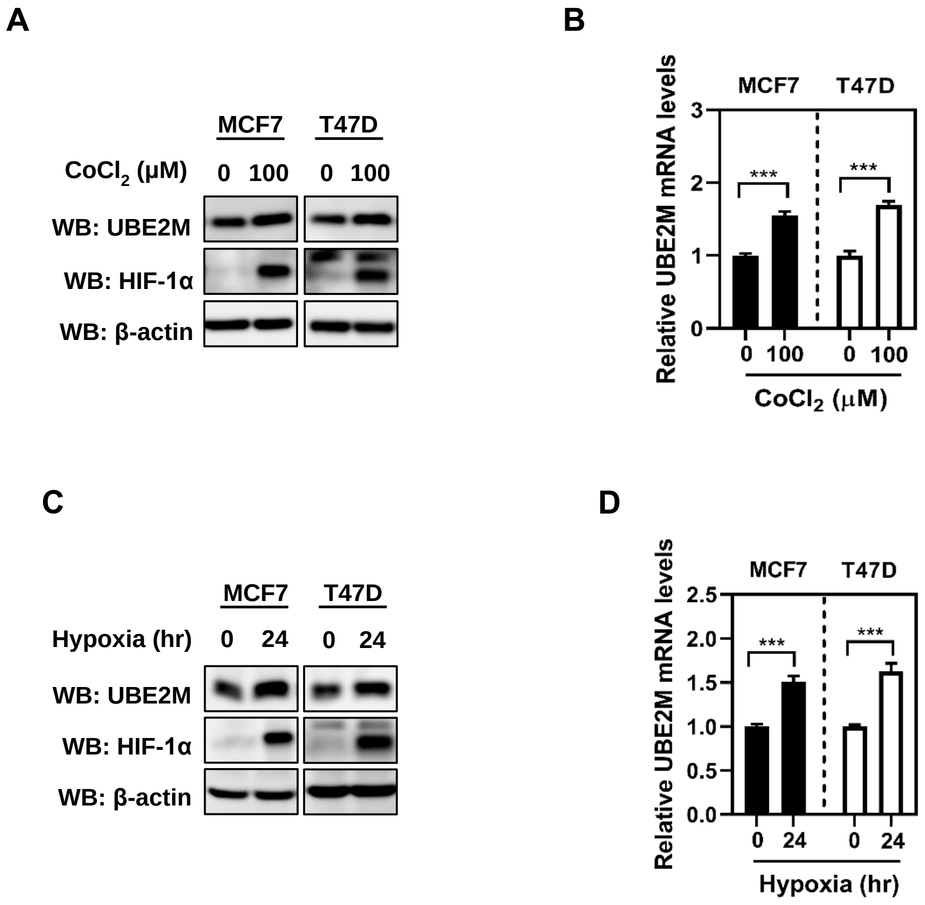


**Figure S5. Hypoxia ($CoCl_2$ treatment or incubation in a hypoxic chamber) led to the accumulation of UBE2M at both protein and mRNA levels.**

# Lin et al. Supplemental Figure 6

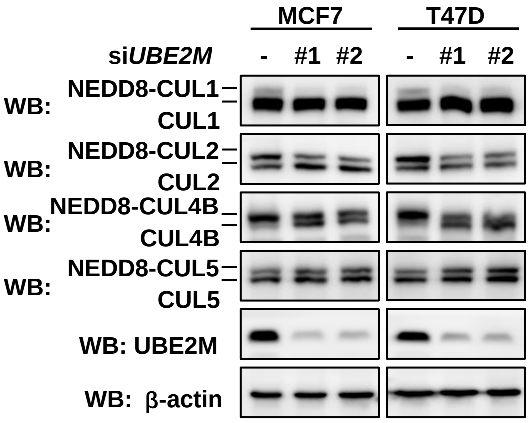


**Figure S6. The effect of UBE2M silencing on neddylation levels of CULs.**

# Lin et al. Supplemental Figure 7

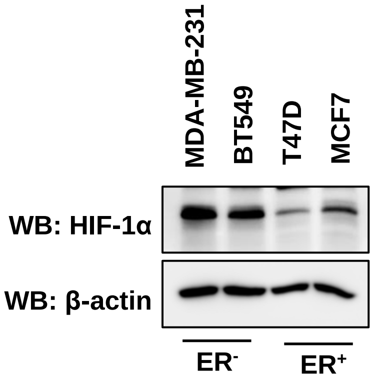


**Figure S7. The expression of HIF-1α in ER$^+$ and ER$^-$ breast cancer cell lines.**

## Lin et al. Supplemental Figure 8

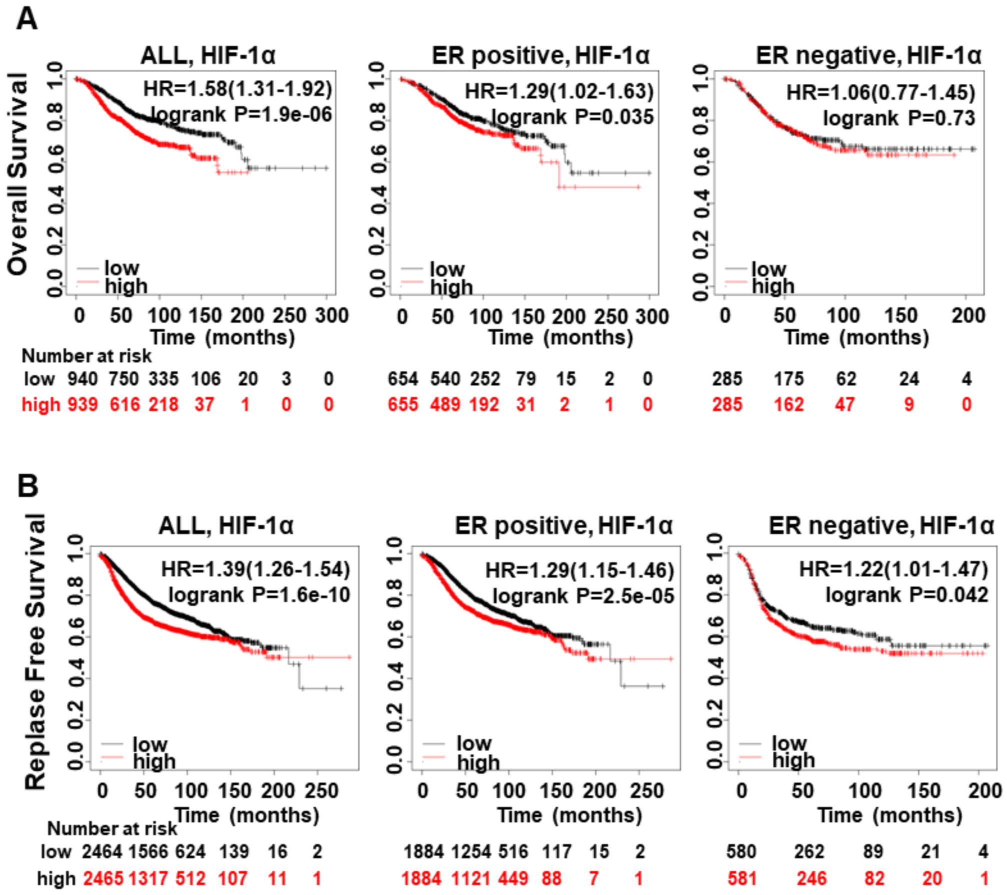


**Figure S8. Correlation between HIF-1α expression and overall survival and relapse-free survival in breast cancer patients with different ER status.** Patients were divided into high group (high) and low group (low) according to the median of UBE2M expression. Number at risk refers to the count of individuals who are still survival at that time point.

# Supplementary Materials and Methods

## Cell culture

The human breast cell lines MCF7, T47D and MDA-MB-231 were obtained from the National Collections of Authenticated Cell Cultures, while the human breast cancer cell line BT549 and the human renal epithelial cell line 293T were obtained from the American Type Culture Collection. MCF7, T47D and 293T cell lines were cultured in Dulbecco's modified Eagle's medium (BasalMedia, L110KJ). MDA-MB-231 was cultured in Leibovitz's L-15 medium (BasalMedia, L620KJ). BT549 was cultured in RPMI-1640 medium (BasalMedia, L210KJ). All media were supplemented with 10% foetal bovine serum (ExCell Bio, FSP500) and 1% penicillin-streptomycin solution (BasalMedia, S110JV). All cell lines were passaged at least five to six times before use, confirmed to be mycoplasma-free, and maintained in a humidified incubator at 37℃ (MDA-MB-231: 100% air; the other cells: 95% air + 5% carbon dioxide).

## Antibodies

Antibodies against BIM (Cell Signaling Technology, 2933, 1:1000 dilution), BRCA1 (Proteintech, 22362-1-AP, 1:1000 dilution), CHIP (Proteintech, 55430-1-AP, 1:1000 dilution), C-PARP (Cell Signaling Technology, 5625, 1:1000 dilution), CTSD (Proteintech, 21327-1-AP, 1:1000 dilution), Cullin1 (Abcam, 75817, 1:1000 dilution), Cullin2 (abcam, 166917, 1:1000 dilution), Cullin3 (Cell Signaling Technology, 2759, 1:1000 dilution), Cullin4A (Cell Signaling Technology, 2699, 1:1000 dilution), Cullin4B (Proteintech, 12916-1-AP, 1:1000 dilution), Cullin5 (Abcam, 184177, 1:1000

dilution), E6AP (Cell Signaling Technology, 7526, 1:1000 dilution), ERα (Cell Signaling Technology, 8644, WB:1:1000 dilution; IP: 1:500 dilution; ChIP: 1:100 dilution), HIF-1α (Cell Signaling Technology, 14179, WB: 1:1000 dilution; ChIP: 1:100 dilution), NOXA (Cell Signaling Technology, 14766, 1:1000 dilution), p21 (Cell Signaling Technology, 2947, 1:1000 dilution), PGR (Cell Signaling Technology, 8757, 1:1000 dilution), p27 (Cell Signaling Technology, 3686, 1:1000 dilution), SKP2 (Proteintech, 15010-1-AP, 1:1000 dilution), UBE2M (Abcam, 109507, 1:1000 dilution), and WEE1 (Cell Signaling Technology, 13084, 1:1000 dilution) were purchased commercially.

**Analysis of RNA-sequencing data from a TCGA dataset**

The mRNA transcriptome data in TPM format were obtained from the The Cancer Genome Atlas (TCGA) database (https: //portal.gdc.cancer.gov/). 113 pairs of normal tissues and breast cancer tissues were used for UBE2M expression analysis. We performed the Wilcoxon signed-rank test to compare the expression levels between the paired samples of cancer and normal tissues. This was done using the wilcox.test function in R, specifying paired = TRUE to indicate a paired test. Adjusted *p*-values were calculated based on the p-values to control the False Discovery Rate (FDR) using the Benjamini-Hochberg (BH) method, implemented via the *p*. adjust function with the method set to "BH". Data was shown in Supplementary Table 3.

**Kaplan-Meier survival analysis**

Kaplan Meier survival curve analysis is performed by the online website Kaplan-

Meier plotter (https://kmplot.com/analysis/). Breast cancer gene-chip data set was selected for analysis. Patients were grouped by median and ER status were selected in the ER status-array.

**Gene silencing using small interfering RNAs**

The cells were transfected with small interfering RNA (siRNA) oligonucleotides using Lipofectamine RNAiMAX (Invitrogen, USA). Cells were seeded to be 60% density at transfection (using a well/6-well plate as an example). 3 μL Lipofectamine RNAiMAX Reagent and 3 μL of 20 μM siRNA oligos were diluted in 200 μL Opti-MEM medium (Gibco, 31985070), respectively. Subsequently, the diluted siRNA was combined with the diluted Lipofectamine RNAiMAX Reagent, and the siRNA-lipid complex was added to the cells after a 15-minute incubation. Cells were collected 72 hours after transfection. The oligonucleotides were synthesized by GenePharma (Shanghai, China), and their sequences were as follows:

Nontarget control (NC): 5′-UUCUCCGAACGUGUCACGU-3′;

si*ESR1*#1: 5′-GCAAGUUGAUCUUAGUUAAGU-3′;

si*ESR1*#2: 5′-GGGCUCUACUUCAUCGCAU-3′;

si*UBE2M*#1: 5′-GGGCUUCUACAAGAGUGGGAAGUUU-3′;

si*UBE2M*#2: 5′-ACUCCAUAAUUUAUGGCCUGCAGUA-3′;

si*CUL1*: 5'-CUAGAUACAAGAUUAUACAUGCGG-3';

si*CUL2*: 5'-GCACAAUGCCCUUAUUCAA-3';

si*CUL3*: 5'-TTGACGTGAACTGACATCCACATTC-3';

si*CUL4A*: 5'-GAAGAUUAACACGUGCUGGTT-3';

si*CUL4B*: 5'-AAGCCUAAAUUACCAGAAA-3';

si*CUL5*: 5'-CUACUGACUCUGAGAAAUA-3'.

**Real-time reverse transcription polymerase chain reaction**

Total RNA was extracted from cells using an RNA purification kit (EZBioscience, B004D). The reverse transcription reaction was performed on 1 μg of total RNA per sample using the PrimeScript™ RT Reagent Kit (TaKaRa, RR047A). Subsequently, real-time polymerase chain reaction (RT-PCR) was performed using TB Green® Premix Ex Taq™ (TaKaRa, RR420A) on the ABI StepOne Plus thermocycler (Thermo Fisher Scientific, USA) according to the instrument manual. Data were analyzed using the ΔΔCt method. The endogenous control transcripts β-actin were used for normalization. The primers used for PCR were synthesized by Sangon Biotech (Sangon Biotech, China), and their sequences were as follows:

Human *β-actin* forward: 5´-TCCCTGGAGAAGAGCTACG-3´;

Human *β-actin* reverse: 5´-GTAGTTTCGTGGATGCCACA-3´;

Human *UBE2M* forward: 5´-ATGAGGGCTTCTACAAGAGTGG-3´;

Human *UBE2M* reverse: 5´- ATTGTCTCACACTTCACCTTGG-3´;

Human *HIF-1α* forward: 5´- GAACGTCGAAAAGAAAAGTCTCG-3´;

Human *HIF-1α* reverse: 5′-CCTTATCAAGATGCGAACTCACA-3′.

**Hypoxia treatment**

Cells (80% density in 6 cm dish) were incubated in a hypoxia incubator (Healforce, HF100 Tri-gas, China) or treated with 100 μM $CoCl_2$ (Selleck, S9490) for 24 hours, and collected for western blotting or RT-PCR. Parameters of hypoxia incubator: 1% $O_2$, 94% $N_2$ and 5% $CO_2$, 37℃.

**Protein half-life measurement**

Cells were transfected or treated under indicated conditions, followed by treatment with 50 μg/mL cycloheximide (CHX, Selleck, S7418) for 0, 2, 4, 8 hours, and collected for western blotting. Protein expression was further quantified using ImageQuant TL. The relative protein expression levels of ERα or UBE2M were determined by normalizing the targeted protein to β-actin.

**MG132 treatment**

UBE2M-silenced cells were treated with 10 μM proteasome inhibitor MG132 (Selleck, S2619) for 6 hours, and collected for western blotting. Protein expression was further quantified using ImageQuant TL. The relative protein expression levels of ERα were determined by normalizing the targeted protein to β-actin.

**Immunoprecipitation and western blotting**

For anti-ERα immunoprecipitation, MCF7 cells were lysed in ice-cold RIPA buffer (Beyotime, P0013D) supplemented with protease inhibitors (Beyotime, P1005 and

ST507). Cell lysates were clarified by centrifugation at 12,000 rpm for 15 minutes. Supernatants were collected and incubated with anti-ERα antibody (1:500 dilution) at 4 °C overnight with rotation. The following day, cell lysates were incubated with protein A-agarose (1:100 dilution, Santa Cruz, sc-2001) for 2 hours, washed thrice with ice-cold RIPA buffer and analyzed via SDS-PAGE and western blotting.

Western blotting was performed according to the standard procedure. Briefly, cells or IP products were added with SDS-PAGE sample loading buffer (Beyotime, P0015A), heated at 100°C for 10 minutes, and clarified by centrifugation. The same amount of protein was separated by SDS-PAGE. Protein was transferred to 0.2 mm polyvinylidene difluoride membranes (Millipore, ISEQ00010). Five percent nonfat milk in TBST (TBS with 0.1% Tween 20) was used for blocking and dilution of primary and second antibodies. Membranes were blocked at room temperature for 1 hour and incubated with primary antibody overnight at 4 °C. Secondary antibodies (anti-mouse: Proteintech, RGAM001, 1:3000 dilution; anti-rabbit: BIOKE, BK-R05, 1:3000 dilution) were applied after washing and incubated at room temperature for 1 hour. Membranes were then washed thrice with TBST, and signals were developed with a Western ECL kit (Epizyme, SQ201). Western blot images were captured by Amersham Imager 680 (GE Healthcare, USA). Protein expression was further quantified using ImageQuant TL. The relative protein expression levels of targeted proteins were determined by normalizing the targeted protein to β-actin.
