# Supplementary material for: UBE2M forms a positive feedback loop with estrogen receptor to drive breast cancer progression and drug resistance
Source: Cell Death Dis. 2024 Aug 13;15(8):590. doi: 10.1038/s41419-024-06979-x (PMC11322533; doi:10.1038/s41419-024-06979-x)
Supplement: Supplementary file 6 — Original Western Blots [file 41419_2024_6979_MOESM6_ESM.pdf]

Full and uncropped western blot for Figure 1A

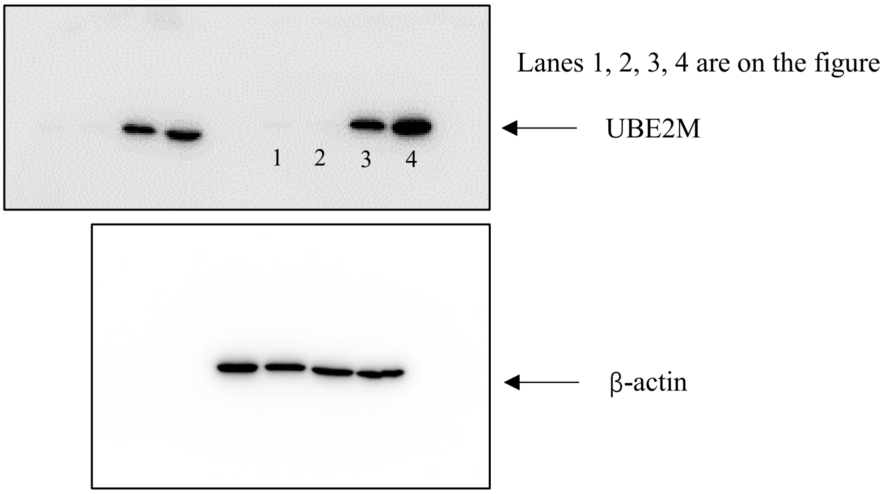

Full and uncropped western blot for Figure 2A

Lanes 1, 2, 3 are on the figure

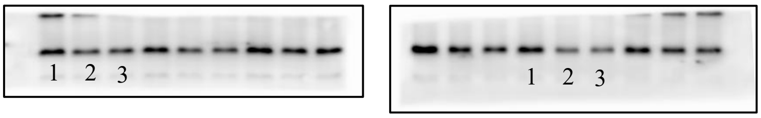

UBE2M

Lanes 1, 2, 3 are on the figure

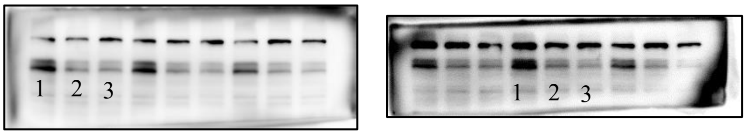

HIF-1α

Lanes 1, 2, 3 are on the figure

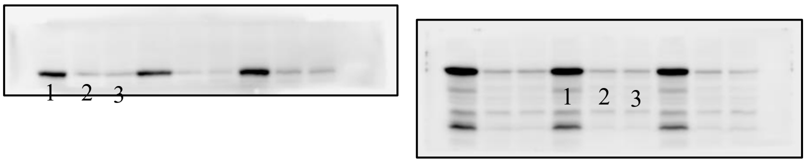

ERα

Lanes 1, 2, 3 are on the figure

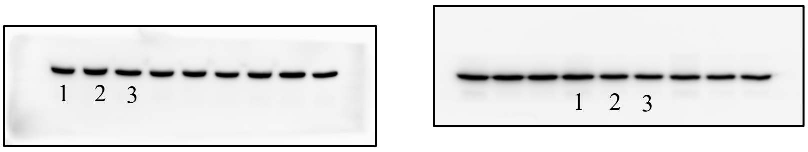

β-actin

Full and uncropped western blot for Figure 2B

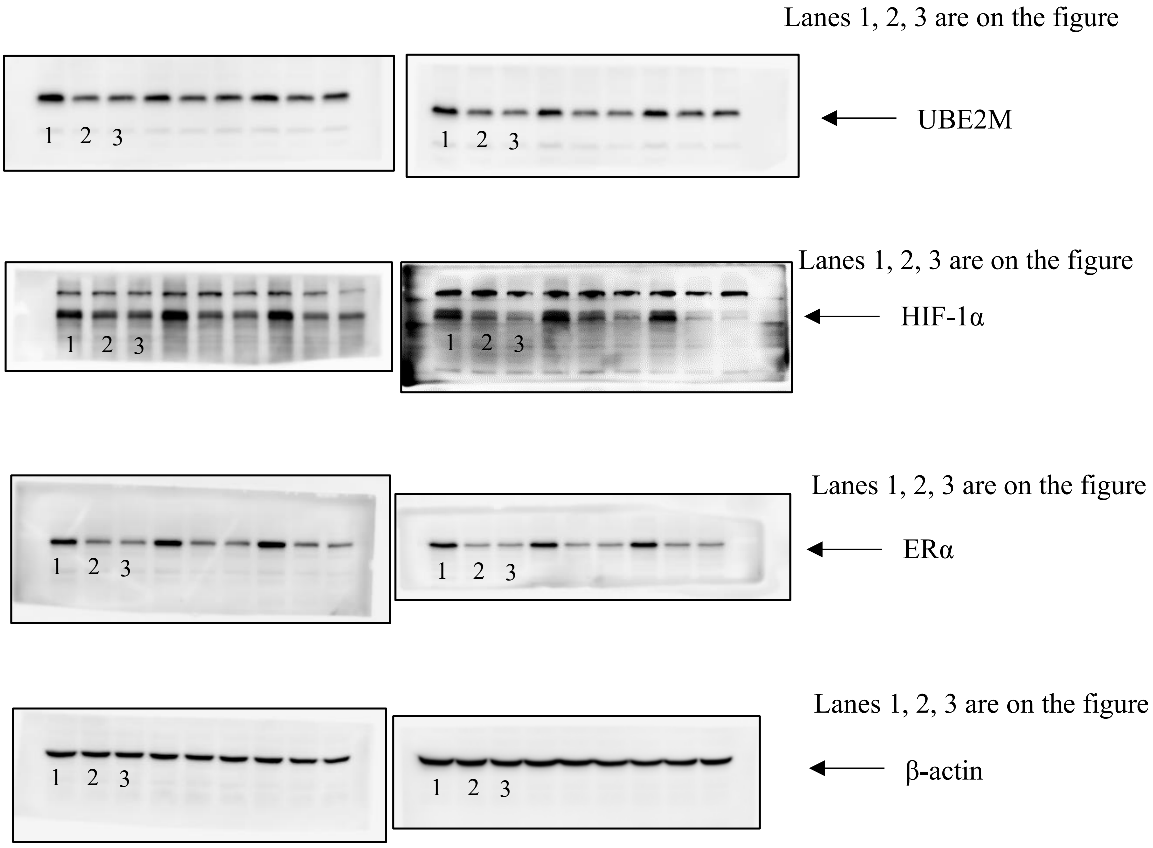

Full and uncropped western blot for Figure 2E

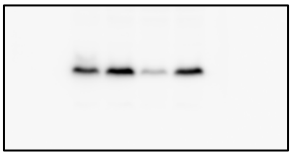

UBE2M

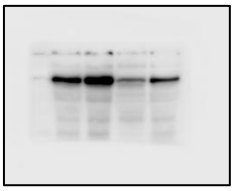

ERα

Lanes 1, 2, 3, 4 are on the figure

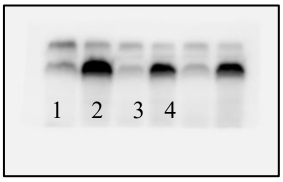

HIF-1α

Lanes 1, 2, 3, 4 are on the figure

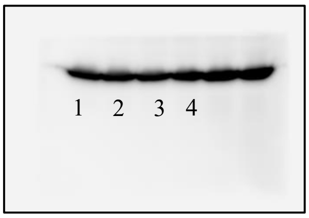

β-actin

Full and uncropped western blot for Figure 2F

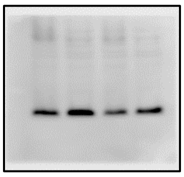

UBE2M

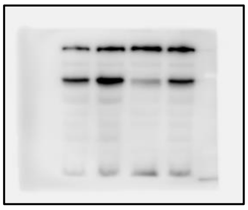

ERα

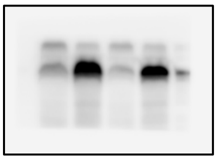

HIF-1α

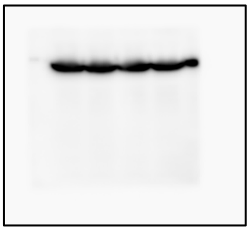

β-actin

Full and uncropped western blot for Figure 3A

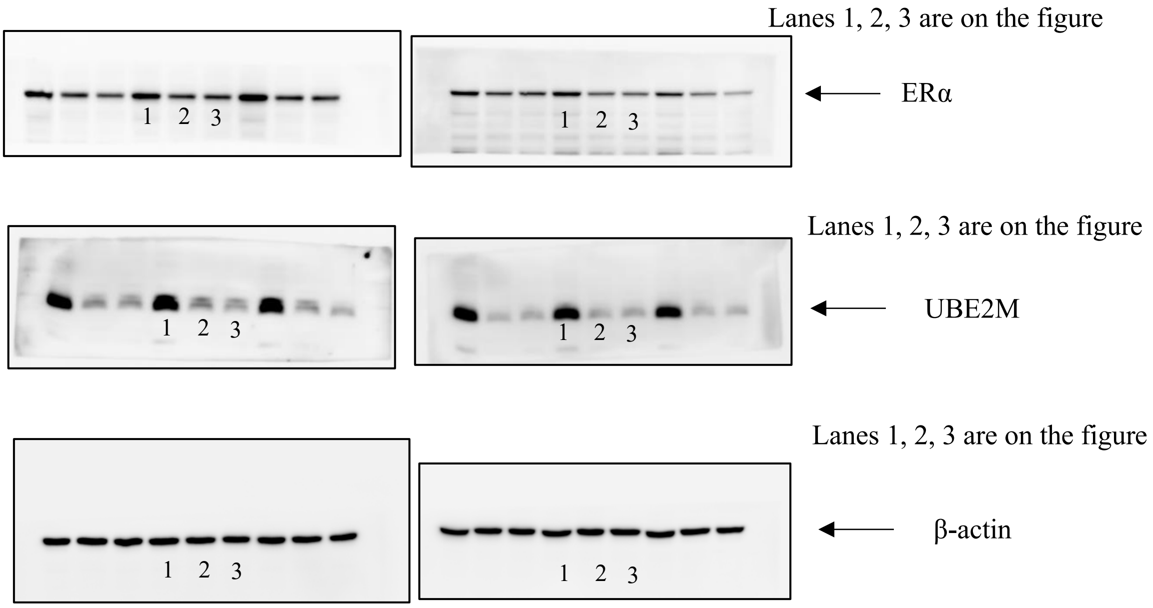

Full and uncropped western blot for Figure 3B

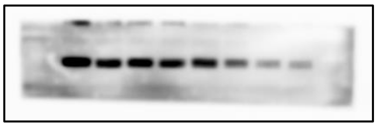

ERα

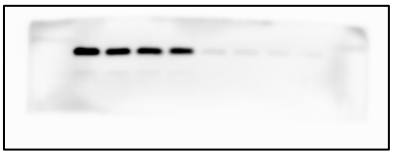

UBE2M

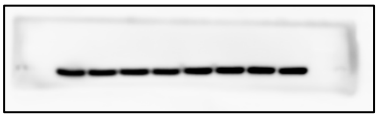

β-actin

Full and uncropped western blot for Figure 3C

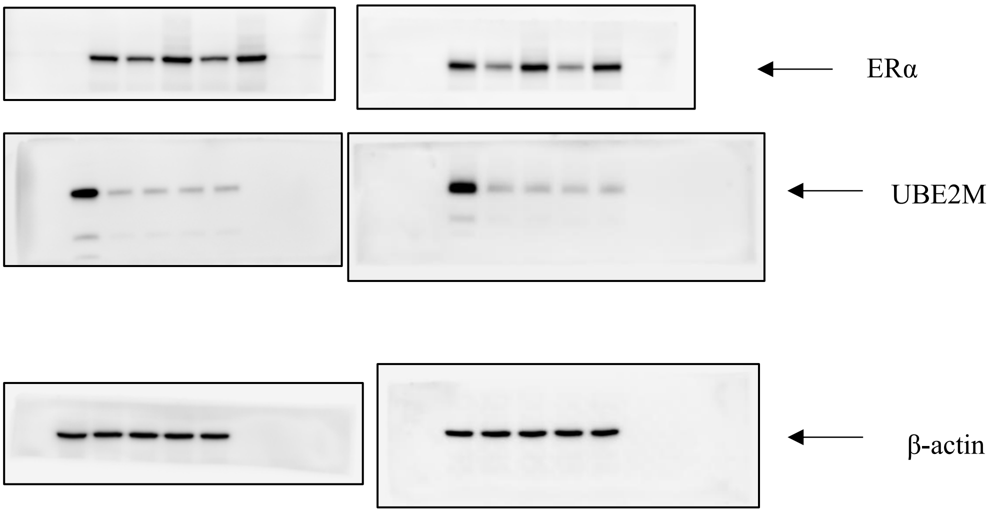

Full and uncropped western blot for Figure 3D

Lanes 1, 2, 3 are on the figure

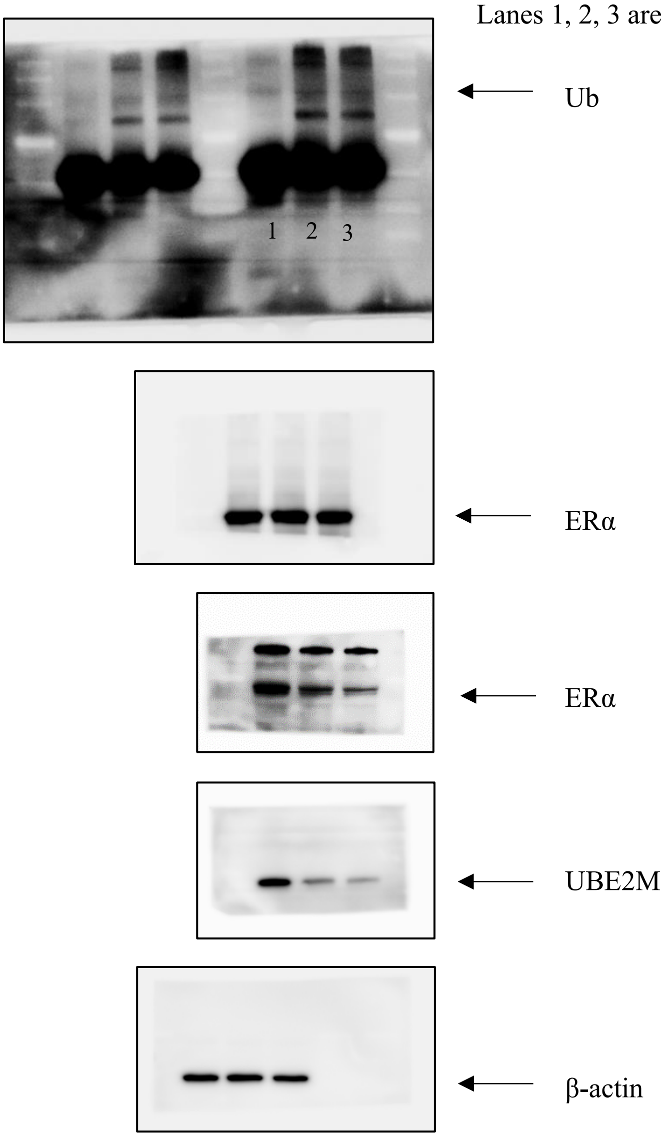

Full and uncropped western blot for Figure 3E

Lanes 1, 2, 3 are on the figure

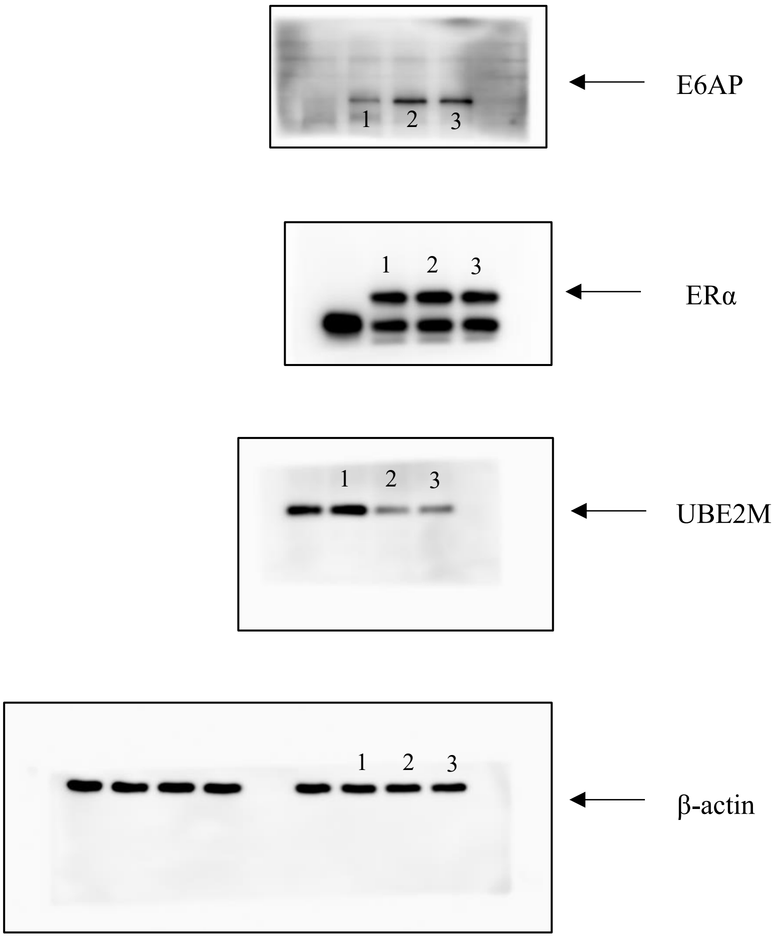

Full and uncropped western blot for Figure 3F

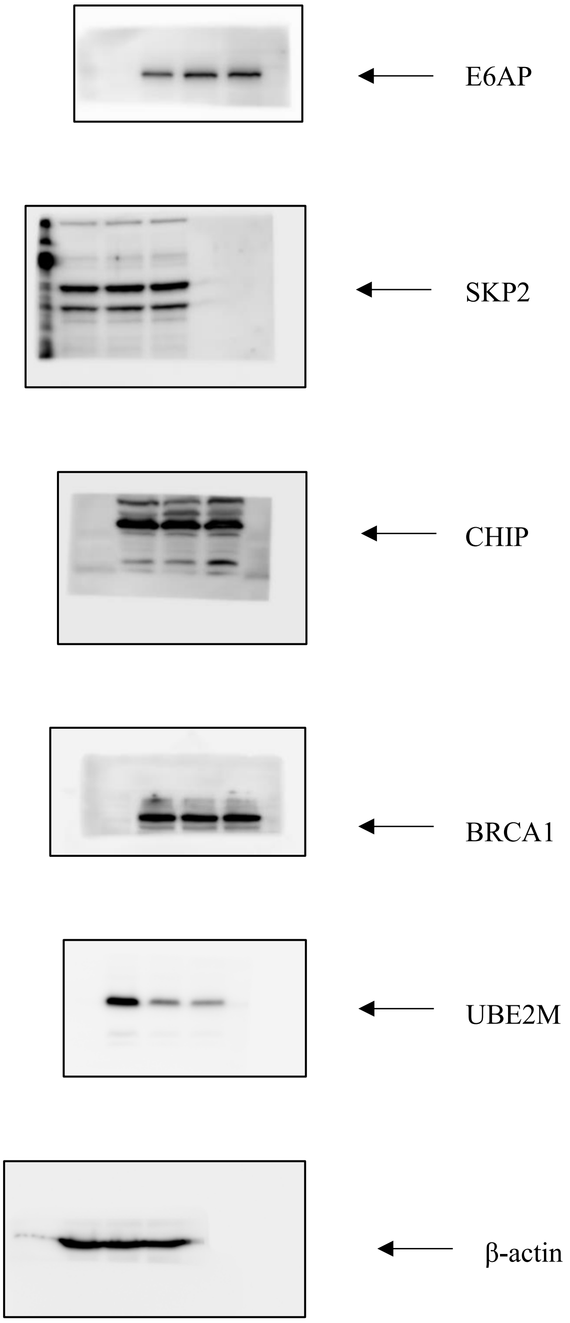

Full and uncropped western blot for Figure 3G

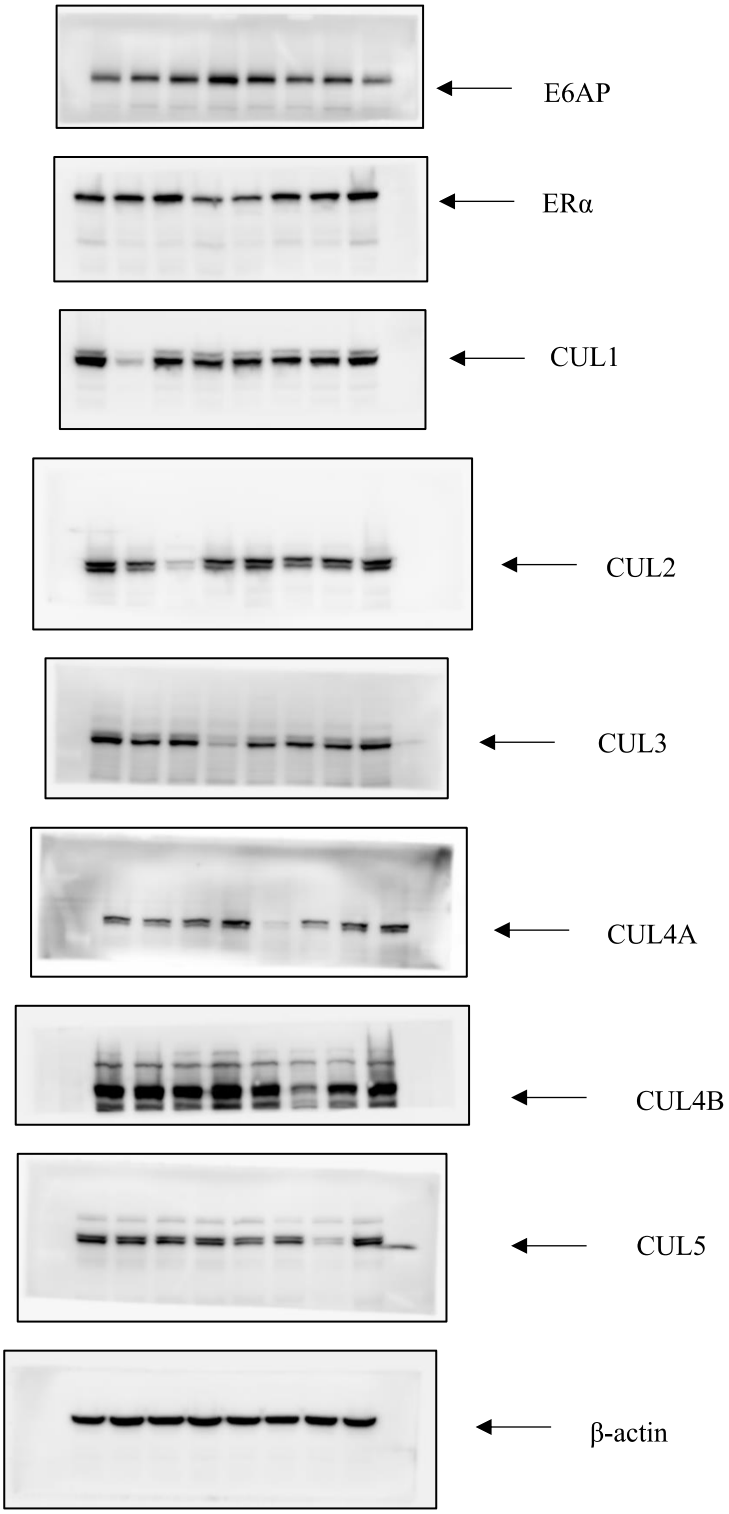

Full and uncropped western blot for Figure 3H

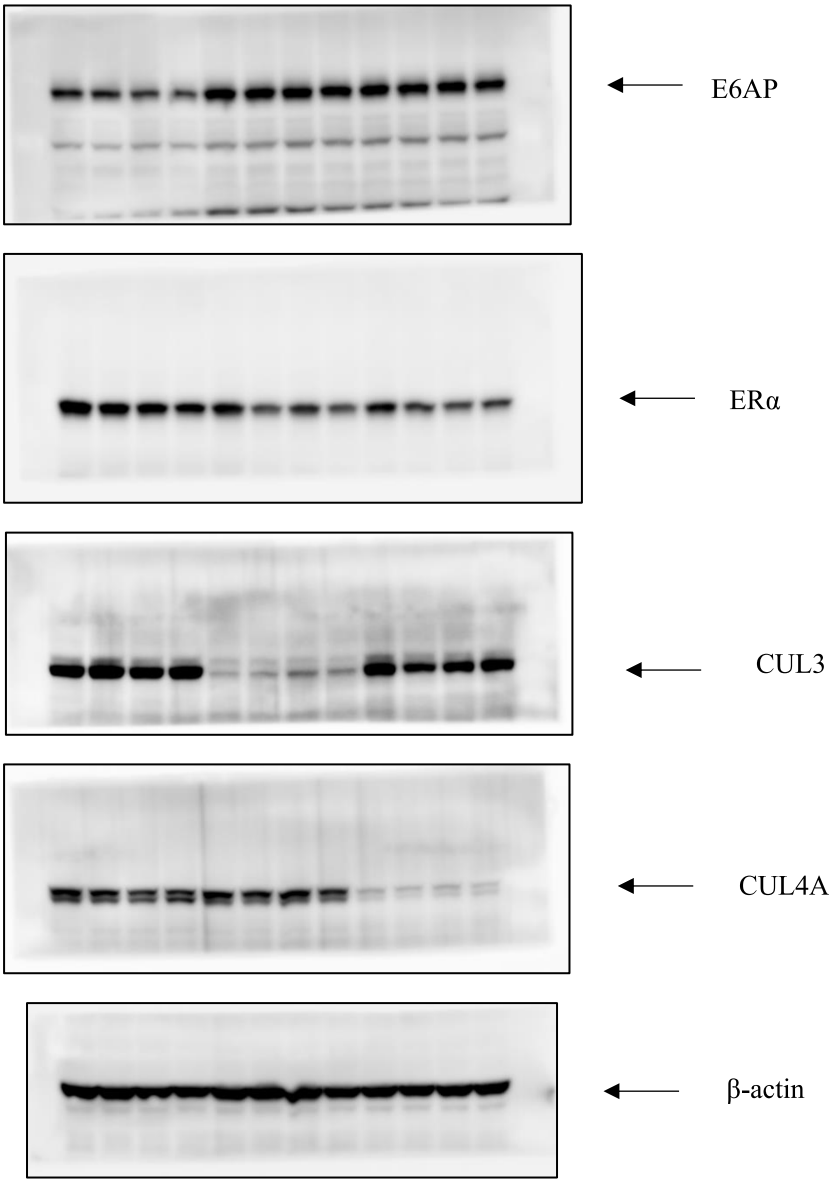

Full and uncropped western blot for Figure 3I

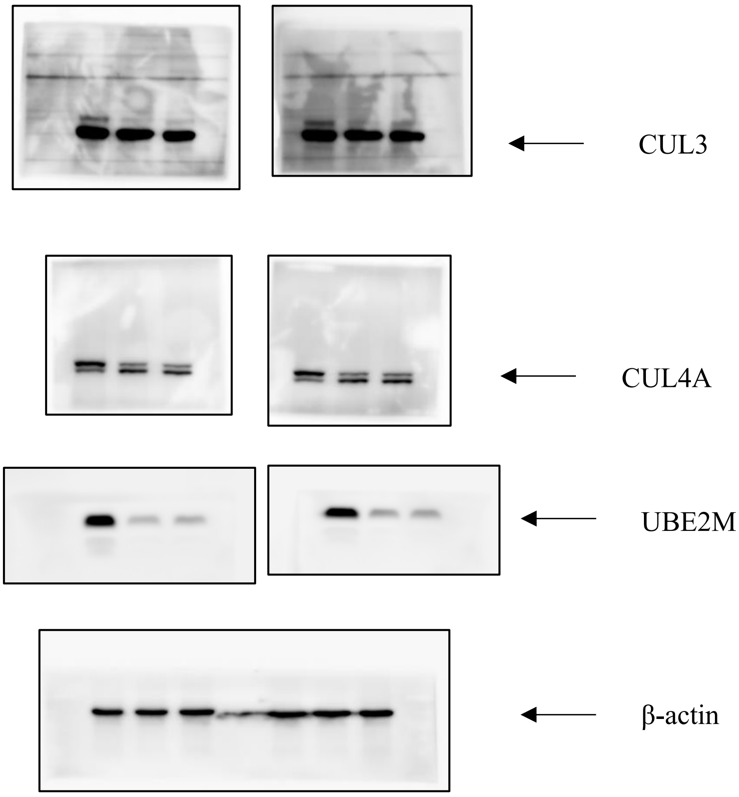

Full and uncropped western blot for Figure 4A

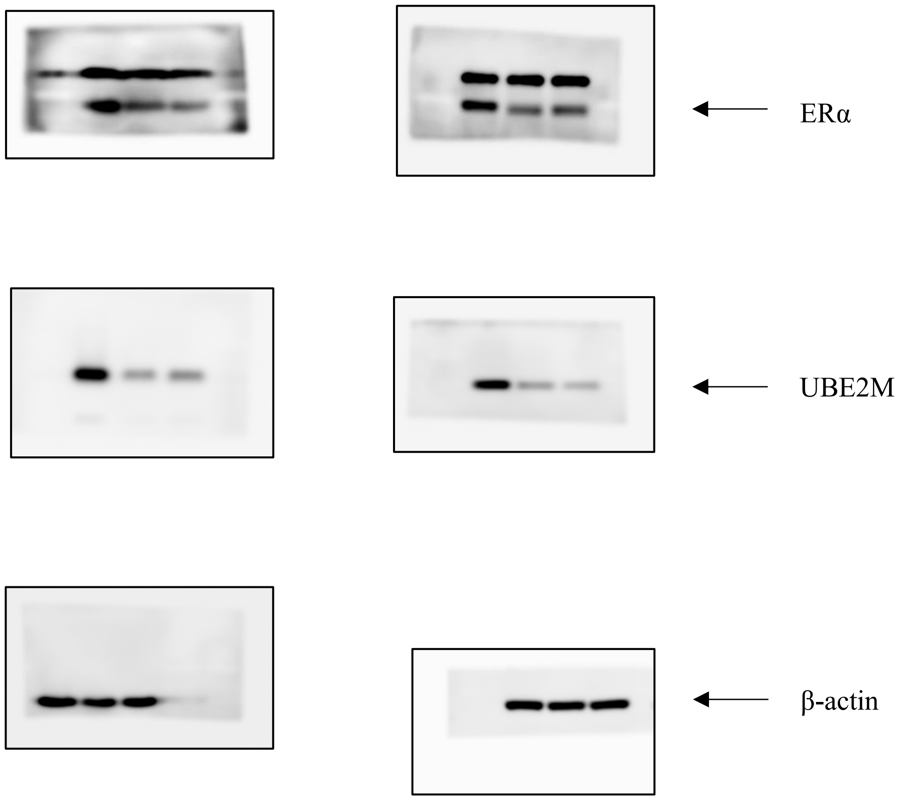

Full and uncropped western blot for Figure 4G

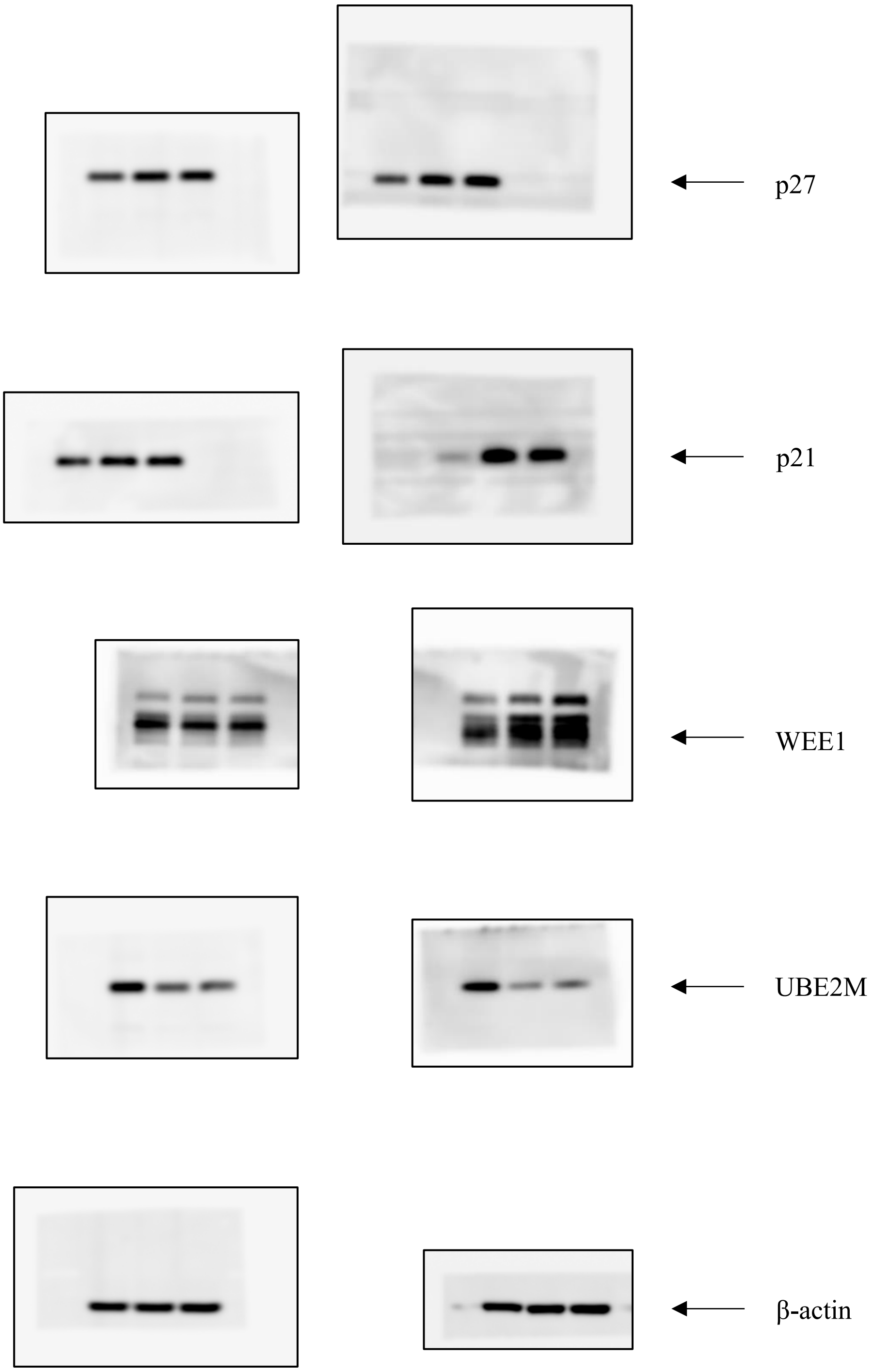

Full and uncropped western blot for Figure 4I

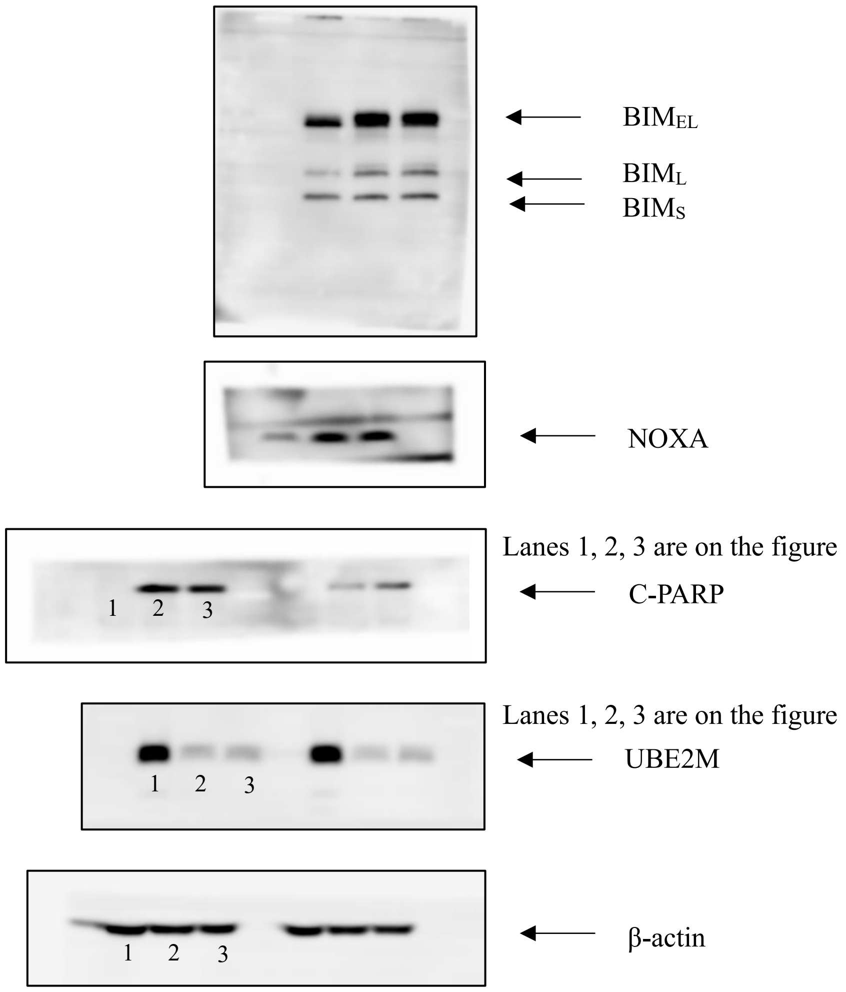

Full and uncropped western blot for Figure 4I

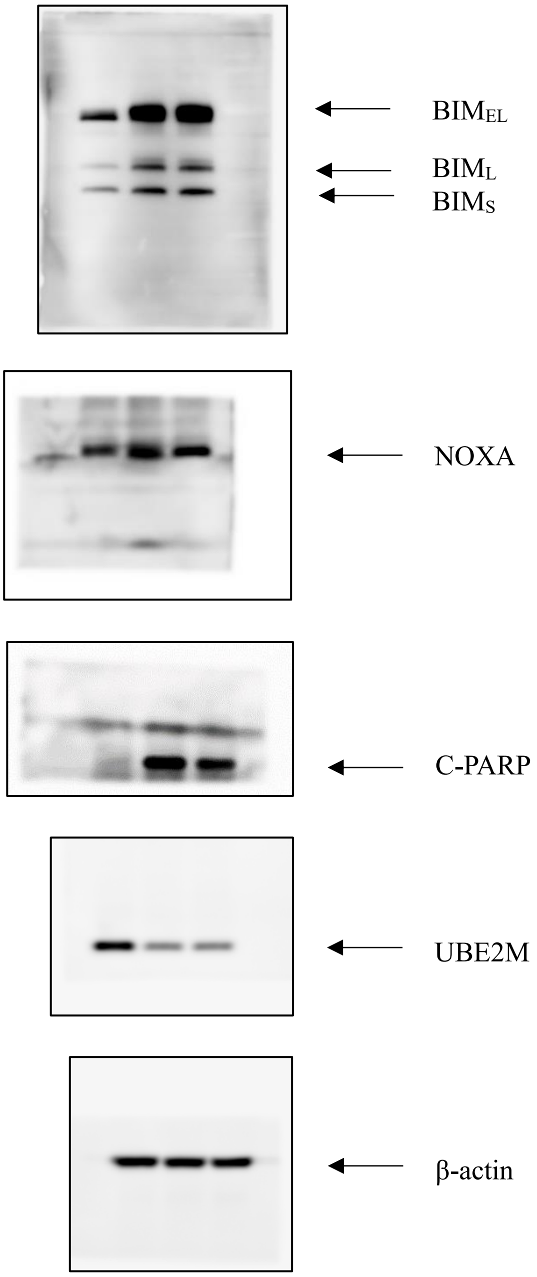

Full and uncropped western blot for Figure 5A

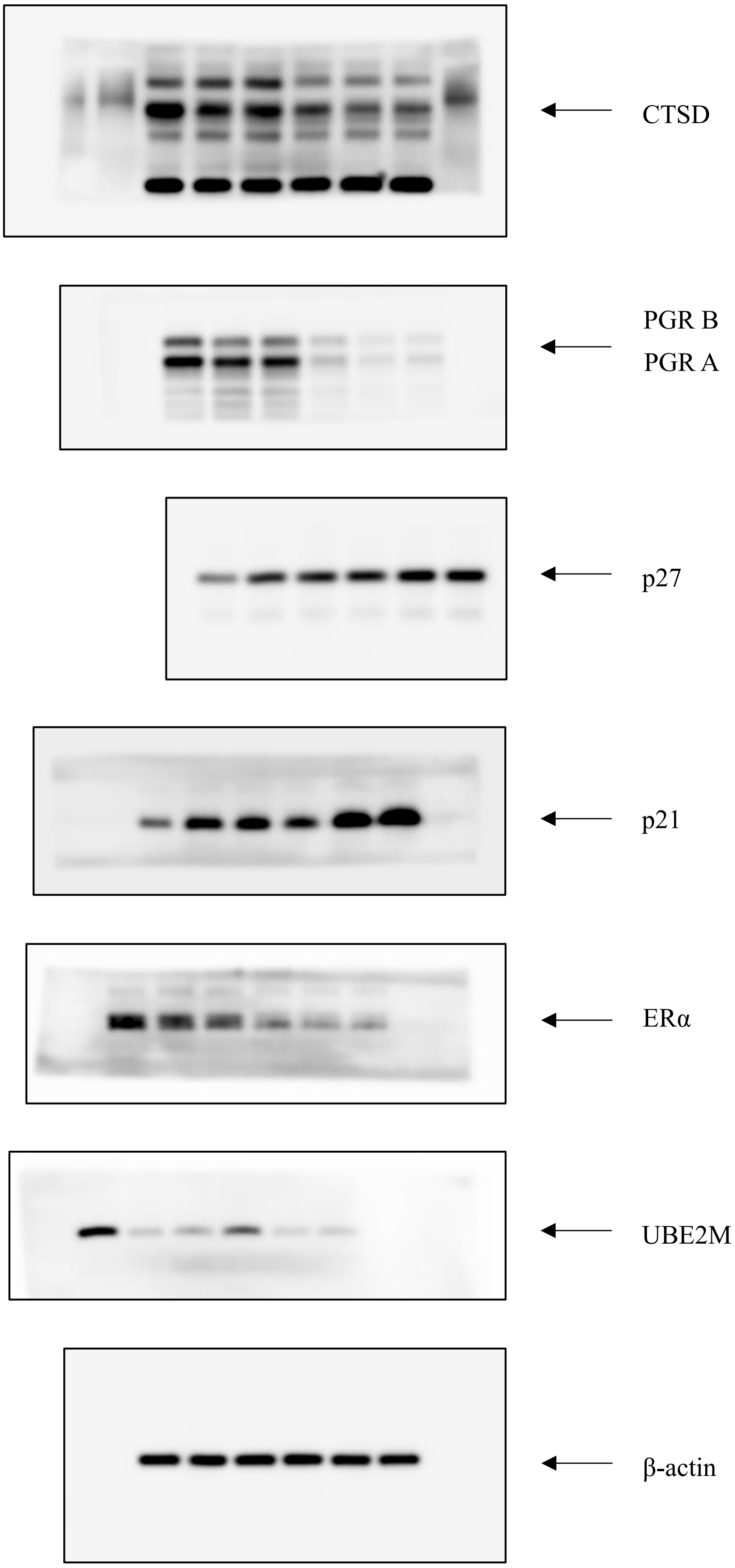

Full and uncropped western blot for Figure 5B

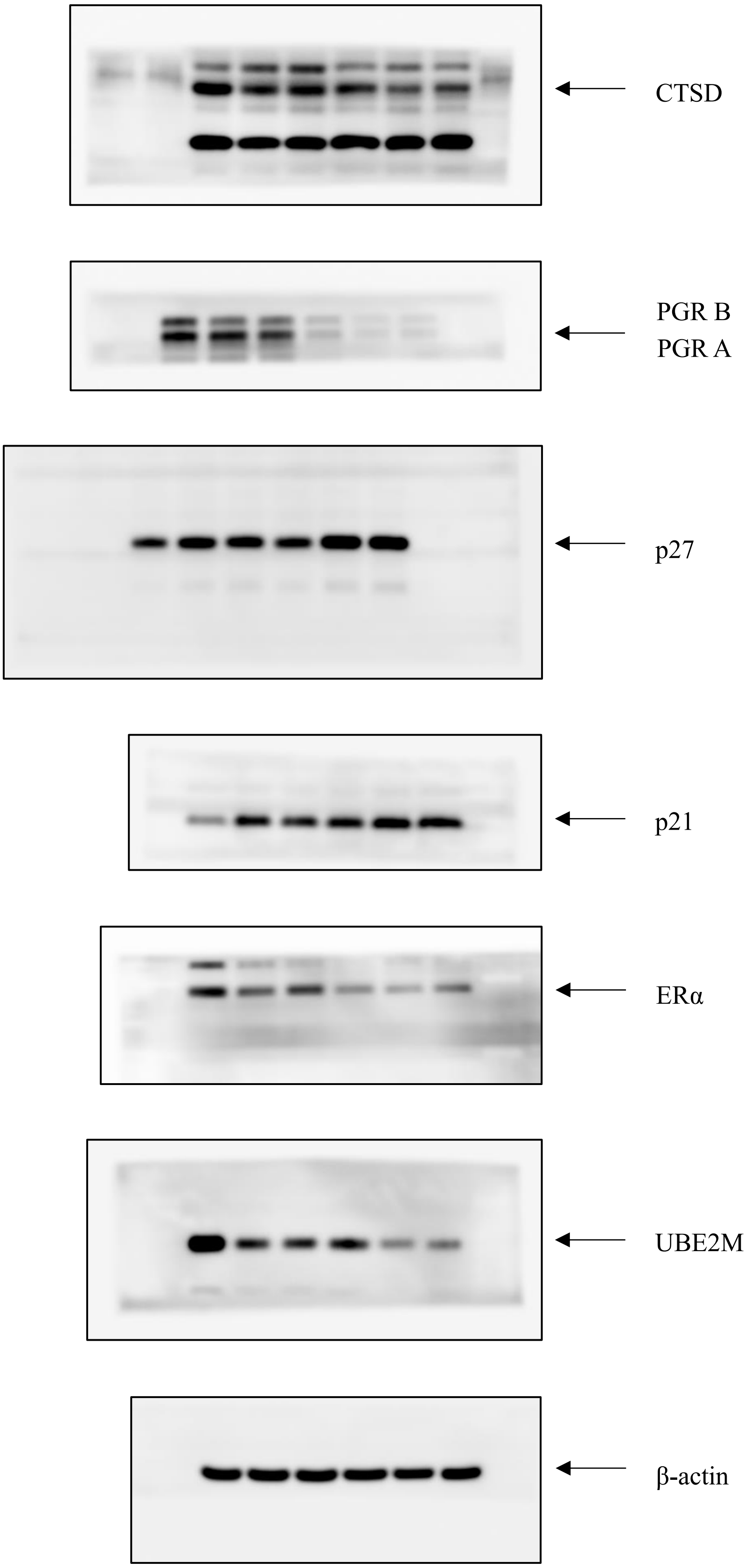

Full and uncropped western blot for Figure 6E

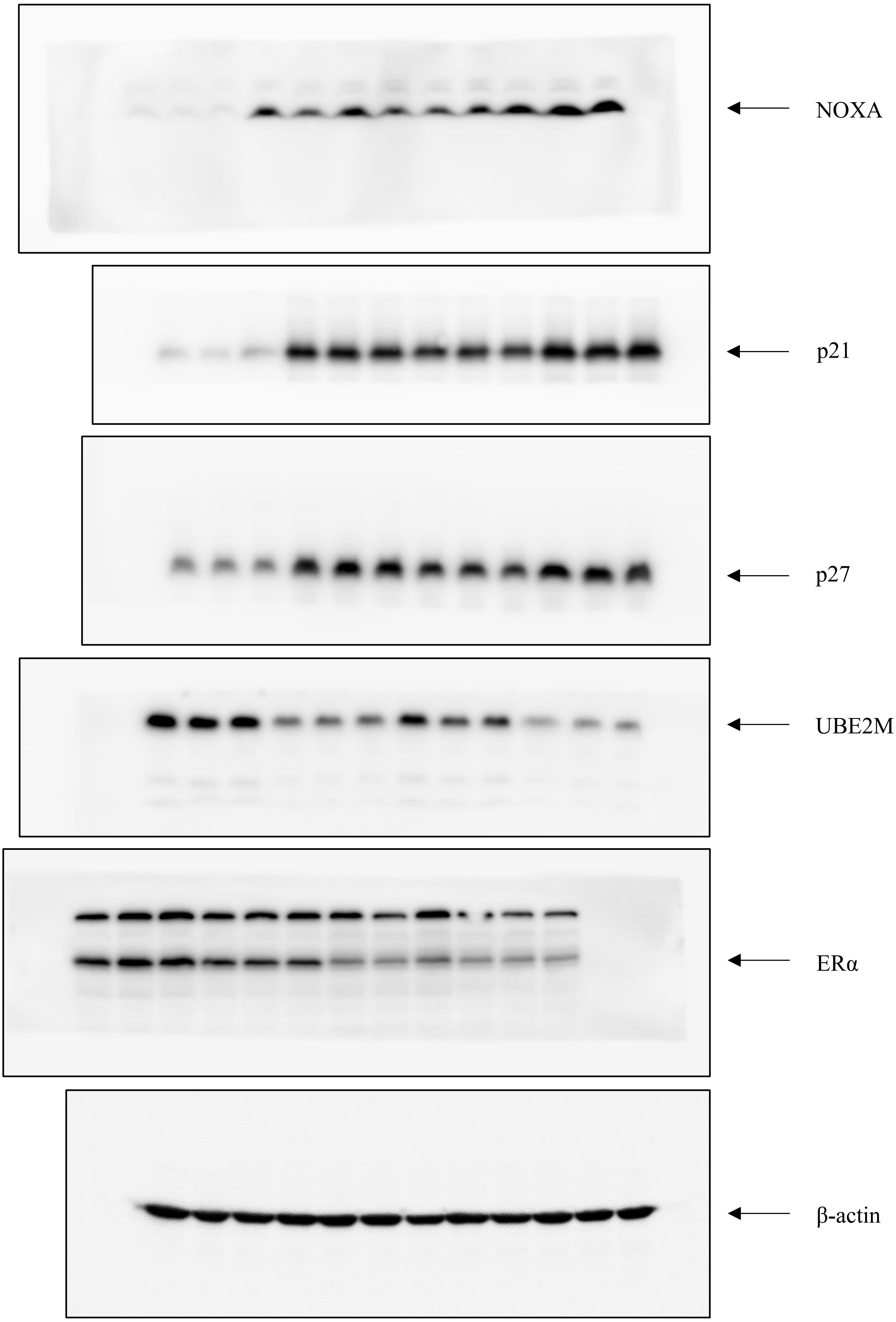

Full and uncropped western blot for Supplemental Figure 4A

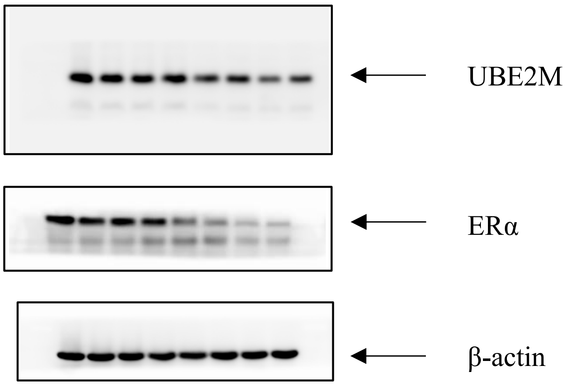

Full and uncropped western blot for Supplemental Figure 4B

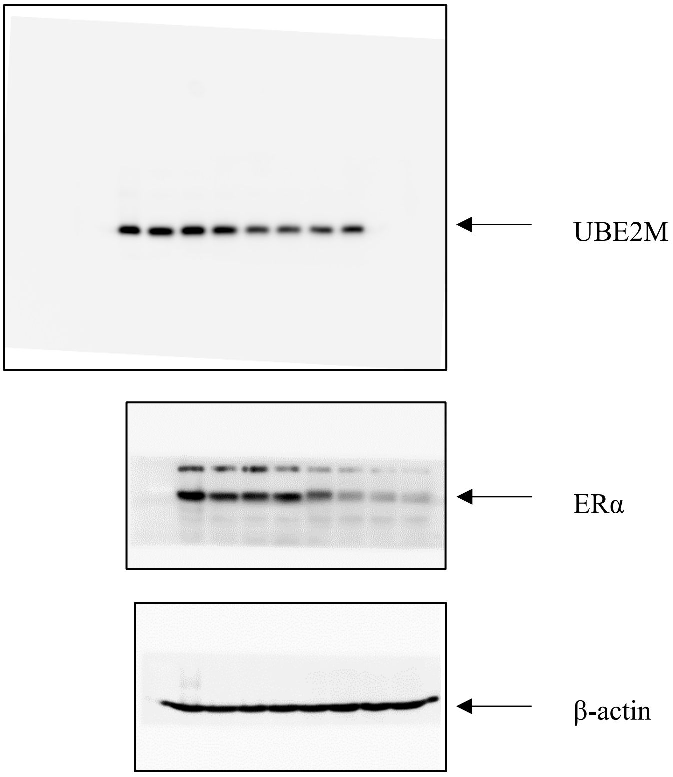

Full and uncropped western blot for Supplemental Figure 5A

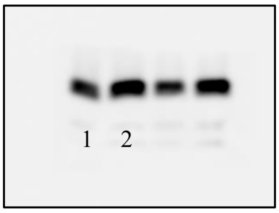

Lanes 1, 2 are on the figure

UBE2M

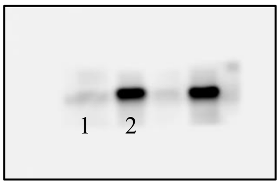

Lanes 1, 2 are on the figure

HIF-1α

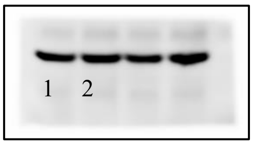

Lanes 1, 2 are on the figure

β-actin

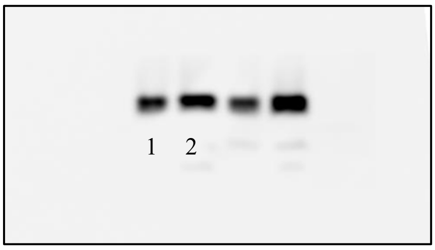

Lanes 1, 2 are on the figure

UBE2M

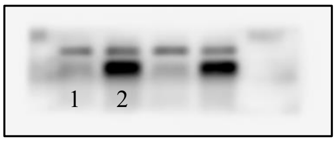

Lanes 1, 2 are on the figure

HIF-1α

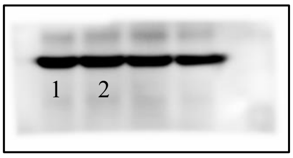

Lanes 1, 2 are on the figure

β-actin

Full and uncropped western blot for Supplemental Figure 5C

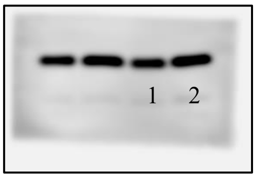

Lanes 1, 2 are on the figure

UBE2M

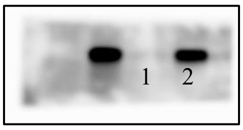

Lanes 1, 2 are on the figure

HIF-1α

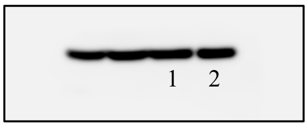

Lanes 1, 2 are on the figure

β-actin

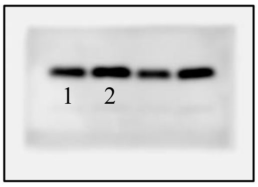

Lanes 1, 2 are on the figure

UBE2M

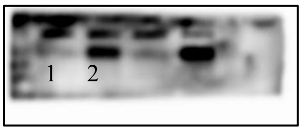

Lanes 1, 2 are on the figure

HIF-1α

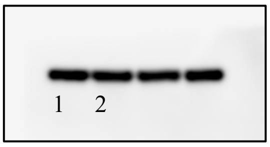

Lanes 1, 2 are on the figure

β-actin

Full and uncropped western blot for Supplemental Figure 6

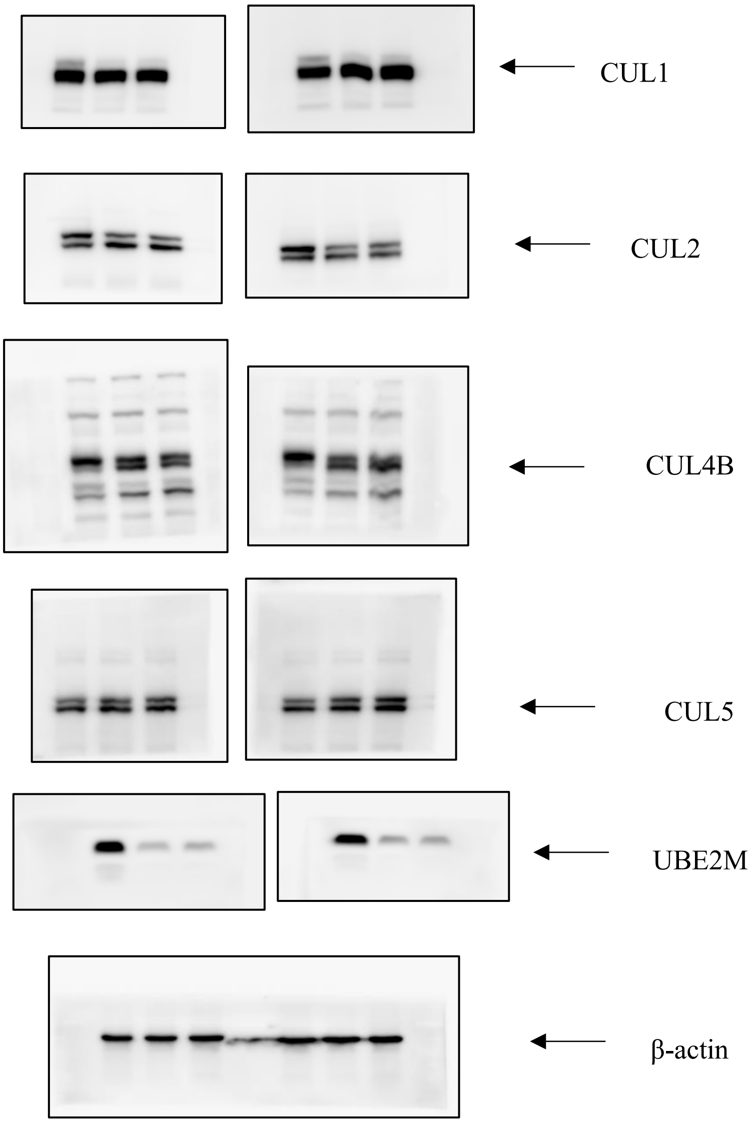

Full and uncropped western blot for Supplemental Figure 7

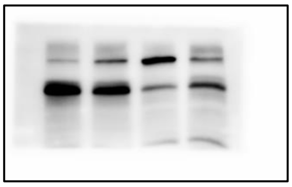

HIF-1α

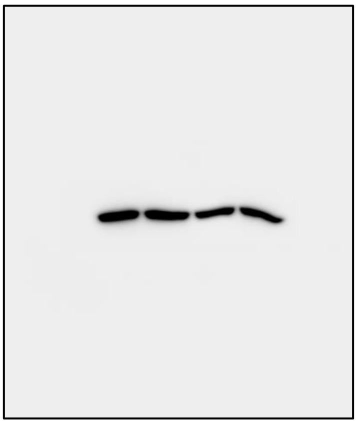

β-actin
